# Identification and Characterization of Two Aryl Sulfotransferases from Deep-Sea Marine Fungi and Their Implications in the Sulfation of Secondary Metabolites

**DOI:** 10.3390/md22120572

**Published:** 2024-12-20

**Authors:** Nicolas Graziano, Beatriz Arce-López, Tristan Barbeyron, Ludovic Delage, Elise Gerometta, Catherine Roullier, Gaëtan Burgaud, Elisabeth Poirier, Laure Martinelli, Jean-Luc Jany, Nolwenn Hymery, Laurence Meslet-Cladiere

**Affiliations:** 1 Univ Brest, INRAE, Laboratoire Universitaire de Biodiversité et Écologie Microbienne, F-29280 Plouzané, France; nicolas.graziano@univ-brest.fr (N.G.); beatriz.arcelopez@univ-brest.fr (B.A.-L.); gaetan.burgaud@univ-brest.fr (G.B.); elisabeth.poirier@univ-brest.fr (E.P.); jean-luc.jany@univ-brest.fr (J.-L.J.); nolwenn.hymery@univ-brest.fr (N.H.); 2 Laboratory of Integrative Biology of Marine Models (LBI2M), Station Biologique de Roscoff (SBR), CNRS, Sorbonne Université, F-29688 Roscoff, France; barboun@sb-roscoff.fr (T.B.); delage@sb-roscoff.fr (L.D.); 3 Institut des Substances et Organismes de la Mer, Nantes Université, ISOMER, UR 2160, F-44000 Nantes, France; elise.gerometta@univ-nantes.fr (E.G.); catherine.roullier@univ-nantes.fr (C.R.); 4 Department of Biochemistry, Max Planck Institute for Chemical Ecology, Hans-Knöll Strasse 8, 07455 Jena, Germany; martinelli.laure.labo@gmail.com

**Keywords:** marine fungi, enzymatic sulfation, sulfated metabolites, sulfotransferases, cytotoxic activity, sulfated mycotoxin

## Abstract

Sulfation plays a critical role in the biosynthesis of small molecules, regulatory mechanisms such as hormone signaling, and detoxification processes (phase II enzymes). The sulfation reaction is catalyzed by a broad family of enzymes known as sulfotransferases (SULTs), which have been extensively studied in animals due to their medical importance, but also in plant key processes. Despite the identification of some sulfated metabolites in fungi, the mechanisms underlying fungal sulfation remain largely unknown. To address this knowledge gap, we conducted a comprehensive search of available genomes, resulting in the identification of 174 putative SULT genes in the Ascomycota phylum. Phylogenetic analysis and structural modeling revealed that these SULTs belong to the aryl sulfotransferase family, and they are divided into two potential distinct clusters of PAPS-dependent SULTs within the fungal kingdom. SULT genes from two marine fungi isolated from deep-sea hydrothermal vents, *Hortaea werneckii* UBOCC-A-208029 (*Hw*SULT) and *Aspergillus sydowii* UBOCC-A-108050 SULT (*As*SULT), were selected as representatives of each cluster. Recombinant proteins were expressed in *Escherichia coli* and biochemically characterized. *Hw*SULT demonstrated high and versatile activity, while *As*SULT appeared more substrate-specific. Here, *Hw*SULT was used to sulfate the mycotoxin zearalenone, enhancing its cytotoxicity toward healthy feline intestinal cells.

## 1. Introduction

Sulfation, also known as sulfonation, was first identified in the 19th century [1] and has since been recognized as a critical modification involved in a wide array of physiological processes [2]. While extensively studied in animals for its medical importance, sulfation has progressively emerged as a multifaceted process across the tree of life. Numerous molecules contain one or more sulfate groups (–SO_3_), spanning from specialized metabolites to macromolecules essential for cellular structure such as glycosaminoglycans and polysaccharides. Sulfation is catalyzed by specialized enzymes called sulfotransferases, which ensure the transfer of a sulfate group from a donor molecule to a hydroxyl (-OH) or an amine (-NH_2_) group on the acceptor substrate. Eukaryotic organisms universally utilize 3′-phosphoadenosine 5′-phosphosulfate (PAPS) as the sulfate donor [2], resulting in the conservation of key regions within all PAPS-dependent sulfotransferases. These conserved regions, including the well-characterized 5′PhosphoSulfate-Binding (5′PSB) and 3′Phosphate-Binding (3′PB) motifs, form the PAPS-binding pocket, which is essential for the correct positioning and transfer of the sulfate group [2,3].

Sulfotransferases are generally classified into cytosolic and membrane-associated types, with the cytosolic sulfotransferases commonly referred to as SULTs. A more refined classification now subdivides sulfotransferases into multiple families based on their biochemical and structural properties, facilitating the identification and analysis of such enzymes across multiple databases (e.g., UniProt, Pfam, InterPro). In the Metazoa kingdom, SULTs contribute to several crucial physiological processes. Among the biotransformation pathways, SULTs are important phase II enzymes (catalyzing conjugation reactions) involved in detoxification by increasing the solubility of endogenous or exogenous compounds or reducing their bioactivity [2]. Conversely, sulfation may lead to the bioactivation of some exogenous compounds such as prodrugs [4,5,6], while also conferring cytotoxic or carcinogenic properties on others [7,8,9,10,11,12]. Additionally, SULTs and sulfatases play non-trivial roles in hormone regulation, where sulfated hormones are rendered inactive, serving as circulatory forms, until sulfatases restore their bioactivity at target organs. The disruption of this balance is associated with several pathologies [13,14].

In the Viridiplantae kingdom, numerous SULT genes have been identified, and their diversity appears significant, although less studied compared to their animal counterparts [15]. While the comprehensive biological roles of plant SULTs (commonly called SOTs) are not fully understood, numerous studies have highlighted their involvement in many physiological processes, such as growth, development, and defense against biotic and abiotic stresses [16,17,18]. Beyond their physiological roles, several of these sulfated plant metabolites exhibit various bioactivities, including antimicrobial, anti-inflammatory, antioxidant, and even antitumor properties [19,20].

In the Fungi kingdom, knowledge of sulfation mechanisms remains largely unexplored. Although fungi are taxonomically and metabolically rich, only a single study has demonstrated the existence of a PAPS-dependent sulfotransferase from the terrestrial fungus *Fusarium graminearum* PH-1, capable of sulfating polyketide-type secondary metabolites [21]. Fungi are among the most versatile and adaptable eukaryotic organisms, inhabiting virtually every ecological niche, whether marine, lacustrine, or terrestrial [22]. With approximately 155,000 recognized species (COL | The Catalogue Of Life, [23]), fungi represent a vast reservoir of biodiversity, and estimates suggest the global richness of fungal species may range from 2.2 million to as many as 19 million [24,25,26]. Fungi play a vital role in maintaining ecological dynamics and stability. Through their various lifestyles (e.g., saprophytic, symbiotic, parasitic), they are involved in processes such as nutrient cycling, organic matter decomposition, and population regulation [27]. Fungal biodiversity is reflected in a rich secondary metabolism, a key factor in their adaptability and ecological success. Fungal secondary metabolites are a major source of new drugs, many of which are now used in human pharmacopeia, thanks to their diverse and varied bioactivities [28]. Examples include antimicrobial drugs like penicillin [29], cephalosporin [30], and griseofulvin [31]; immunosuppressants such as cyclosporine [32] and mycophenolic acid [33]; and cholesterol-lowering lovastatin [34]. Several fungi are a promising source of anticancer drugs [35], such as the marine fungus *Aspergillus sydowii*, which produces diorcinolic acid [36]. However, numerous fungi also produce toxic metabolites, such as mycotoxins (e.g., zearalenone), that can cause acute toxicity, leading to organ damage and toxic syndrome, or long-term toxicity, including carcinogenesis, mutagenesis, and hormonal syndrome [37], leading to public health challenges.

Despite the richness of fungal secondary metabolites, very few sulfated natural compounds have been identified and published, most of which have been isolated from plant-associated or marine-derived fungi. Notable examples include a sulfoalkylresorcinol exhibiting activity against the polymerization of the cell division protein FtsZ along with antibacterial properties, isolated from *Zygosporium* sp. KNC52, a marine fungus isolated from corals [38]. In addition, co-cultivation of *Penicillium crustosum* PRB-2, an Antarctic deep-sea fungus, and *Xylaria* sp. HDN13-249, a marine-derived fungus, produced the sulfated molecules Penixylarin B and 1,3-dihydroxy-5-(12-sulfoxyheptadecyl)benzene, both of which exhibit antibacterial properties [39]. Although these compounds have been chemically characterized, the biosynthetic pathways responsible for their sulfation remain unknown, strengthening the need to delve deeper into the exploration of fungal sulfotransferases.

Here, we contribute to the understanding of fungal SULTs by identifying and characterizing the first two SULTs from marine fungi, *Hortaea werneckii* UBOCC-A-208029 and *Aspergillus sydowii* UBOCC-A-108050, isolated from deep-sea hydrothermal vents [40,41]. Through phylogenetic analysis, structural modeling, and biochemical characterization, we provide new insights into fungal sulfation mechanisms and the biotechnological potential of these SULTs.

## 2. Results

### 2.1. Genome Analysis Reveals Two Phylogenetically Distinct Clusters of Fungal SULTs

**Figure 1:**
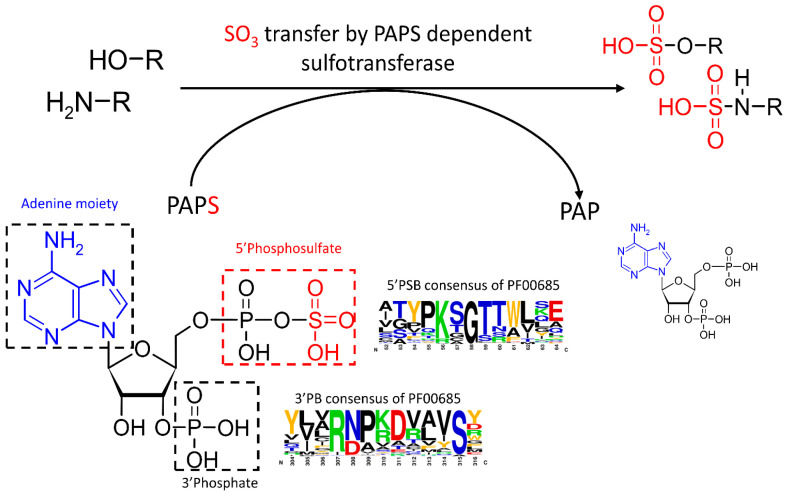
General mechanism of PAPS-dependent sulfotransferase.

The Mycocosm database was queried to identify genes encoding putative SULTs containing the PF00685 Pfam domain (Sulfotransfer_1 domain), associated with PAPS-dependent sulfotransferases (SULTs and carbohydrate sulfotransferases). The Hidden Markov Model (HMM) profile for PF00685 includes highly conserved motifs crucial for PAPS binding, such as 5′PSB and 3′PB (Figure 1). Additionally, the seed alignment used to define PF00685 is mainly composed of well-characterized SULTs, ensuring a robust and reliable identification of the Sulfotranfer_1 domain in previously uncharacterized sequences.

A total of 235 hits were obtained and submitted to InterproScan [42] for a refined analysis based on protein signature detection (e.g., HMM) and sequence homology across multiple databases, including Pfam, InterPro, and Panther. Sixty-one hits were excluded due to ambiguous annotations, insufficient sequence lengths (<150 residues), or the absence of typical PAPS-binding motifs. After manual filtering, a final set of 174 putative fungal SULTs was retained. Although most of the proteins detected came from terrestrial fungal strains, some have been identified within fungi that may have a marine lifestyle, notably *Hortaea werneckii* EXF-2000, isolated from marine Sečovlje salterns (Slovenia) on the Adriatic coast [43] and *Aspergillus sydowii* CBS 593.65, isolated from Caribbean Sea fan corals [44]. Two different strains were available in our marine collection, *H. werneckii* UBOCC-A-208029 and *A. sydowii* UBOCC-108050, both isolated from the deep-sea waters of the Mid-Atlantic Ridge, at the Rainbow site near a hydrothermal vent at a depth of 2300 m. Based on *H. werneckii* EXF-2000 and *A. sydowii* CBS 593.65 SULT sequences, homologous SULT genes were identified in *H. werneckii* UBOCC-A-208029 and *A. sydowii* UBOCC-108050. For convenience, the putative SULT of *H. werneckii* UBOCC-A-208029 was renamed *Hw*SULT, while that of *A. sydowii* UBOCC-1080500 was renamed *As*SULT. *Hw*SULT shares 96.50% sequence identity with the putative SULT from *H. werneckii* EXF-2000. *As*SULT shares 97.99% sequence identity with the putative SULT from *A. sydowii* CBS 593.65. The nucleotide sequences for the *H. werneckii* UBOCC-A-208029 SULT (*Hw*SULT, PQ561061) and the *A. sydowii* UBOCC-108050 SULT (*As*SULT, PQ562869) are available on GenBank database.

**Figure 2:**
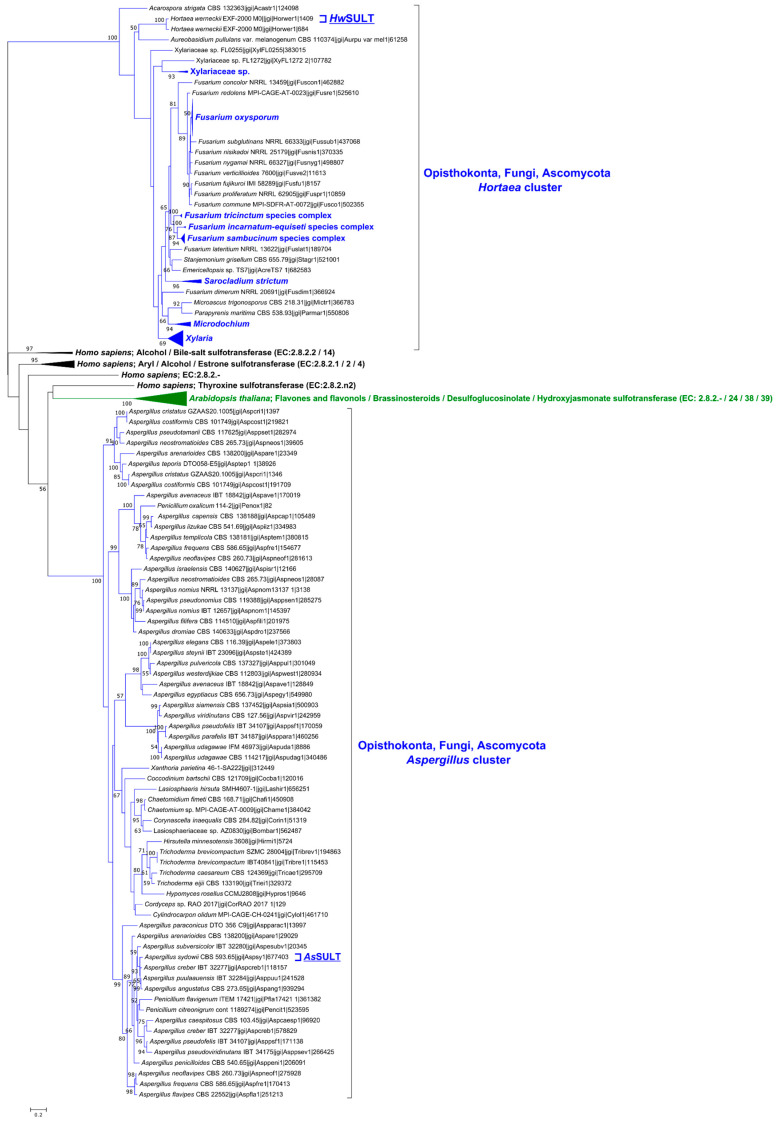
Phylogenetic tree of putative fungal SULTs. Sulfotransferase domains were aligned and phylogeny analyzed by maximum likelihood (RaxMl v. 8.2.12). The reliability of the trees was tested by bootstrap analysis using 100 resamplings of the dataset. Only bootstrap values above 50% are shown. Fungi are colored in dark blue, humans in black, and *A. thaliana* in green. Accession numbers of used proteins are listed in Table S1.

In 2020, Xie et al. [21] performed a phylogenetic study that revealed that the fungal SULTs belonged to the aryl sulfotransferase family (SULT), and they were divided into two well-separated clusters. However, these two clusters emerged from a limited dataset of twelve fungal sequences, which represent only a fraction of the total diversity. The 174 putative fungal SULTs identified in this work could provide a more representative view of fungal SULT diversity. Thus, to explore the diversity, taxonomic distribution, and clustering patterns of the 174 fungal SULTs as well as to assess the reliability of two previously established clusters, a new phylogenetic analysis was conducted, focusing on the residues forming the sulfotransfer_1 domain (PF00685) of all 174 putative fungal SULTs. Sequences from *H. werneckii* EXF-2000 and *A. sydowii* CBS 593.65 were used for phylogenetic analyses, as their genomes are fully sequenced and publicly available, unlike the *H. werneckii* UBOCC-A-208029 and *A. sydowii* UBOCC-108050 strains. Functionally characterized SULTs from humans and from *Arabidopsis thaliana* were added to improve tree structuring and examine relationships with fungal SULTs. The resulting maximum likelihood tree reveals two distinct fungal clusters (Figure 2). These clusters, designated and named according to the enzymes characterized in this study (SULTs from *Aspergillus sydowii* UBOCC-A-108050 and from *Hortaea werneckii* UBOCC-A-208029), corresponded to clusters highlighted by Xie et al., (2020) [21]. The *Aspergillus* cluster and the *Hortaea* cluster, which contain 66 and 108 sequences, respectively, are exclusively composed of fungal proteins and are clearly separated from each other (100% bootstrap support) and from the plant and human SULTs, which belong to other clades.

The *Aspergillus* cluster predominantly includes proteins from Eurotiomycota, which represent 80% of the cluster (e.g., *Aspergillus steynii*, *A. sydowii* or *Penicillium oxalicum*). The remaining 20% consists of proteins from Sordariomycota (e.g., *Cordyceps* sp. or *Trichoderma caesareum*) along with a single representative from Lecanoramycota (*Xanthoria parietina*). The *Hortaea* cluster primarily consists of Sordariomycota, which represents 95% of the cluster, with proteins from Hypocreales (e.g., *Emericellopsis*, *Stanjemonium*, *Sarocladium*, *Fusarium)* and from Xylariales (e.g., *Xylaria arbuscula*, *Parapyrenis maritima*). The genus *Fusarium* (e.g., *Fusarium graminearum Fg*SULT1, *F. jujikuroi*, *F. avenaceum*) accounts for approximately 60% of the *Hortaea* cluster. The remaining 5% corresponds to three proteins from Dothideomycota (*Hortaea werneckii* and *Aureobasidium melagenum*) and one protein from Lecanoramycota (*Acarospora strigata*). Despite the inclusion of numerous new sequences, no new clusters emerged, and the two clusters identified by Xie et al., (2020) are conserved [21].

**Figure 3:**
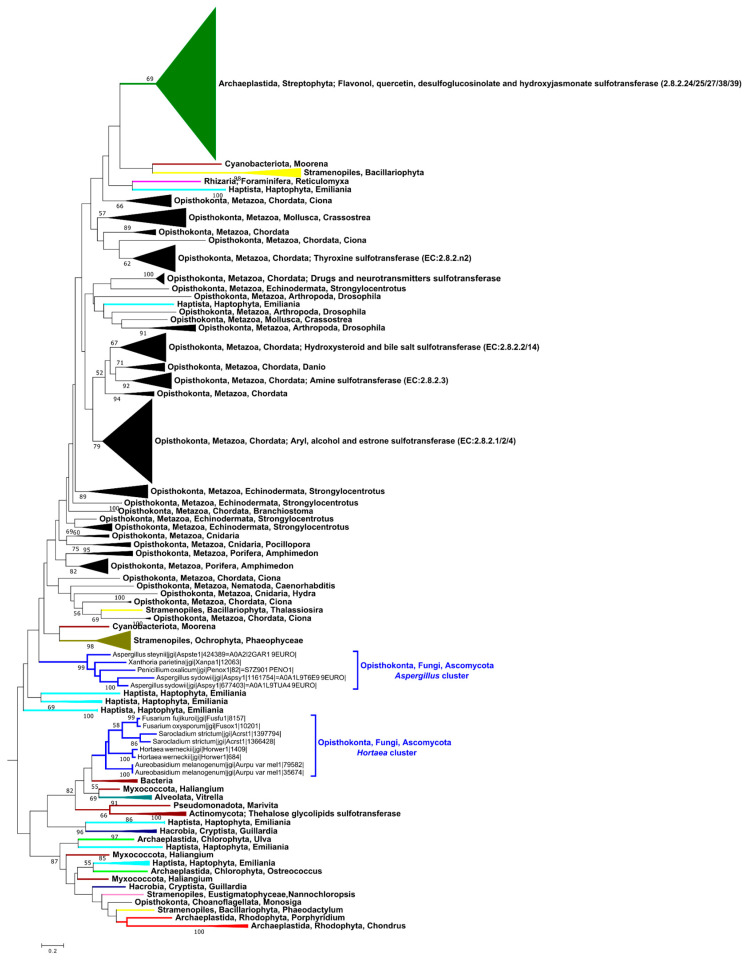
PF00685/St1-1 aryl sulfotransferase (SULT) family phylogenetic tree. Sulfotransferase domains were aligned and manually curated, resulting in 160 informative positions. Phylogeny was analyzed by maximum likelihood (RaxMl v. 8.2.4). The reliability of the trees was tested by bootstrap analysis using 100 resamplings of the dataset. Only bootstrap values above 50% are shown. Fungi are colored in dark blue. Accession numbers of used proteins are listed in Table S2.

While this first analysis provides some hints and confirms the two clusters’ existence, as first observed by Xie et al., (2020) [21], a limited number of non-fungal SULT sequences were included as outgroups (Table S1 and Xie et al., (2020) [21]), which could be considered as insufficient to determine the position of fungal SULTs in a broader evolutionary framework. Consequently, a comprehensive analysis of the “PF00685/aryl sulfotransferase (SULT) family” (Figure 3), which includes 574 proteins from eukaryotic and prokaryotic organisms, was performed.

This dataset features both characterized proteins (e.g., human SULTs, *Arabidopsis thaliana* SOTs) and identified but uncharacterized proteins (e.g., algae). Thirteen representative putative fungal SULTs from the *Aspergillus* and *Hortaea* clusters were included in this analysis. A manual curation strategy was adopted, resulting in 160 informative positions being used to build the maximum likelihood tree. With a significantly higher number of sequences than in previous studies, this analysis extends the investigation of evolutionary relationships to numerous Metazoans, Viridiplantae, Stramenopiles, and various microorganisms (See Table S2). With much more numerous and taxonomically varied sequences, this analysis also confirms the existence of two well-separated fungal clusters and the belonging of these two clusters to the aryl sulfotransferase family. This new analysis also suggests a more distant evolutionary relationship between the *Aspergillus* cluster proteins and human and plant enzymes than previously thought (Figure 2 and study by Xie et al., 2020) [21]. The *Aspergillus* cluster now appears more closely related to marine eukaryotes, in particular brown algae and the alga *Emiliania huxleyi*. The *Hortaea* cluster is nested within a larger, well-supported cluster (82%) mainly composed of diverse bacteria. These vary in many aspects, such as their cell wall composition (Gram-positive and Gram-negative), origin (marine, arid, or tropical soil, animal pathogens), and taxonomy (*Cyanophyceae*, *Myxococcota*, *Gammaproteobacteria*, *Alphaproteobacteria*, and *Actinomycetes*). This largest cluster also includes a marine unicellular alga, *Vitrella brassicaformis.* Despite weak bootstrap support (<50%), three halophilic Gram-negative bacteria isolated from marine environments—*Haliangium ochraceum* (*Myxococcota*), *Gynuella sunshinyii* (*Gammaproteobacteria*), and *Halomicronema hongdechloris* (*Cyanophyceae*)—along with *Vitrella brassicaformis*, appear to exhibit a closer evolutionary relationship with the fungal proteins.

These two phylogenies converged toward two conclusions. All the SULTs identified feature the PF00685 domain and belong to the aryl sulfotransferase family. It could be inferred that Ascomycota does not possess carbohydrate sulfotransferases containing the Pfam PF00685 domain. The total diversity of fungal SULTs is encompassed by two distinct clusters. Although this distribution could indicate significant differences in protein structures and functions, no biochemical information was available for proteins belonging to the *Aspergillus* cluster. To understand the differences between these two clusters, structural investigations and biochemical characterizations were conducted on *Hw*SULT from *H. werneckii* UBOCC-A-208029 and *As*SULT from *A. sydowii* UBOCC-108050.

### 2.2. AsSULT and HwSULT Present a Classic PAPS-Binding Structure

**Figure 4:**
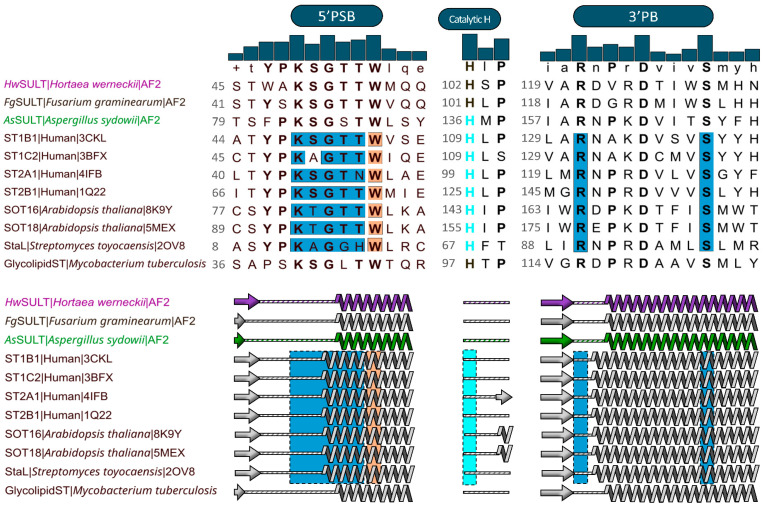
Putative PAPS-binding sites (5′PSB and 3′PB) and catalytic histidine in *As*SULT and *Hw*SULT. Partial alignment was performed with human SULT1B1 (PDB: 3CKL), SULT1C2 (PDB: 3BFX), SULT2A1 (PDB: 4IFB), SULT2B1 (PDB: 1Q22), and *Arabidopsis thaliana* SOT16 (PDB: 8K9Y) and SOT18 (PDB: 5MEX), whose structures were solved in the presence of PAPS. Two prokaryotic SULTs were added, StaL (PDB: 2OV8) from *Streptomyces toyocaensis* NRRL 15009 and the biochemically characterized SULT from *Mycobacterium tuberculosis* H37Rv^T^ (UniProt: P9WGB9). Consensus threshold 70% (bolded residues). Secondary structures derived from PDB files or predicted by AlphaFold2 (AF2) for *Hw*SULT, *Fg*SULT1, *As*SULT, and Glycolipid sulfotransferase. Blue boxes indicate residues involved in hydrogen or ionic bonds with PAPS. Orange boxes indicate residues involved in aromatic–aromatic interaction with adenine moiety of PAPS. Cyan boxes represent catalytic histidine.

The structural features of *As*SULT and *Hw*SULT were explored through multiple sequence alignment (MSA) and structural modeling using AlphaFold2 [45]. *As*SULT and *Hw*SULT were compared to characterized human, plant, and prokaryotic SULTs (SULT1B1, SULT1C2 [46], SULT2A1, SULT2B1 [47], SOT16 [48], SOT18 [49], StaL [50], and Glycolipid sulfotransferase Rv1373 [51]), revealing conserved features typical of PAPS-binding sulfotransferases (Figure 4 and Figure S1). *Hw*SULT and *As*SULT share low primary structure identity, with only 16% of identical residues (Figure S1). *Hw*SULT shares slightly lower sequence identity with plant SULTs (14–15%) and human SULTs (approximately 20%) than *As*SULT, which shares 21% and 21–28% with plants and human SULTs, respectively. Both *Hw*SULT and *As*SULT have very low sequence identity with *Streptomyces toyocaensis* and *Mycobacterium tuberculosis* SULT (14.5% and 12.2%, respectively). Figure 4 depicts the two characteristic PAPS-binding regions identified in *Hw*SULT and *As*SULT, along with a key residue involved in the catalytic process. The first region involved in PAPS binding, i.e., 5′PSB, consists of a KSGTTW region (MSA consensus) responsible for binding the 5′phosphosulfate moiety (KSGTT) and the adenine moiety (W) of the PAPS donor (Figure 1). Structural analysis revealed that this region forms a flexible loop between a β-sheet and an α-helix, as illustrated in Figure 4. In *Hw*SULT and *As*SULT, this region is almost fully conserved at both primary and secondary structure levels (Figure 4).

**Figure 5:**
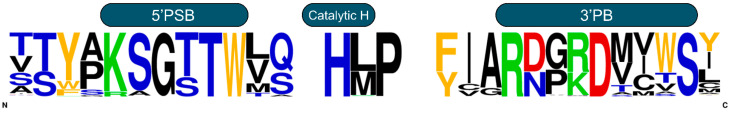
Conservation of 5′PSB, catalytic histidine, and 3′PB among fungal SULTs. The frequency plot is a graphical representation of a multiple sequence alignment of the dataset of 174 sequences. The letter height is proportional to the conservation of the residue.

Considering all putative fungal SULTs (174 sequences), this region also appears to be highly conserved across fungal species (Figure 5). A slight variation occurs between the two enzymes, with threonine at position 52 in *Hw*SULT being replaced by serine at position 86 in *As*SULT, but this variation is likely minor given the similar biochemical properties of threonine and serine. The second region consists of two essential residues, an arginine (121R in *Hw*SULT) and a serine (129S in *Hw*SULT), with seven amino acids between the two residues. These residues are critical as they interact with the 3′phosphate moiety of the donor (Figure 1). In most structures, the arginine is located at the end of β-sheet and the serine, in the middle of the following α-helix, as depicted in Figure 4. These two residues are conserved in *Hw*SULT and *As*SULT, and their secondary structures prediction is concordant with known structures. Interestingly, aspartic acid is fully conserved (125D in *Hw*SULT or 163D in *As*SULT) across all fungal SULTs (Figure 5). This residue appears to stabilize the protein by forming interactions with nearby α-helix residues, suggesting a role in structural integrity. In characterized SULTs, a highly conserved histidine upstream of 3′PB is thought to act as a catalytic base. This positively charged residue is believed to deprotonate the acceptor substrate, thereby facilitating nucleophilic attack of the sulfate group of PAPS [2,52]. This histidine is conserved in both *Hw*SULT and *As*SULT (Figure 4) and is also fully conserved among fungal putative SULTs (Figure 5).

**Figure 6:**
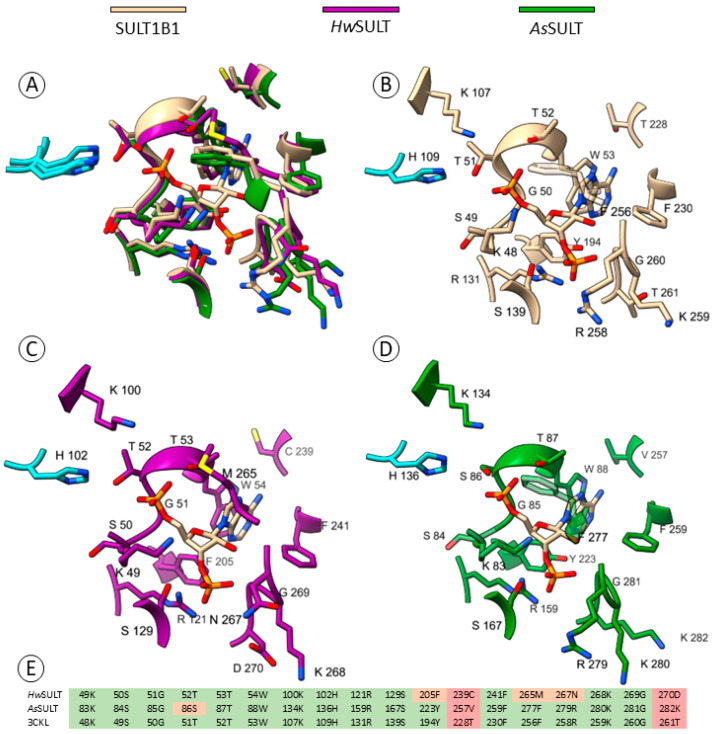
Conservation of the PAPS-binding pocket in *Hw*SULT and *As*SULT. (**A**) Overimposition of PAPS-binding residues in ST1B1 (PDB: 3CKL), *Hw*SULT, and *As*SULT. (**B**) PAPS-binding residues in SULT1B1 (PDB: 3CKL). (**C**) Inferred PAPS-binding residues in *Hw*SULT. (**D**) Inferred PAPS-binding residues in *As*SULT. (**E**) Alignment of PAPS-binding residues. Green boxes indicate conserved residues. Orange boxes indicate partially conserved residues. Red boxes indicate different residues.

Residue analysis across PAPS-binding sites in ST1B1, ST1C2, ST2A1, ST2B1, SOT16, and SOT18, coupled with MSA, identified nine additional putative PAPS-binding residues in *Hw*SULT and *As*SULT. Table S3 summarizes the PAPS-binding residues and Figure S2 shows their sequence identity. Overall, *Hw*SULT and *As*SULT share 67% identity in PAPS-binding regions, with 12 common residues. The putative PAPS-binding residues of *As*SULT exhibit 67% to 83% similarity to human and plant enzymes, while *Hw*SULT shows 61% to 72% similarity. The highest identity was reached for both *As*SULT and *Hw*SULT with human SULT1B1 at 83% and 72%, respectively. To explore the potential three-dimensional structure of *Hw*SULT and *As*SULT, models were generated using AlphaFold2 [45]. Despite the low sequence identity between fungal proteins and characterized SULTs, AlphaFold2 produced high-confidence models due to the structural characterization available for many SULTs. The best model for *As*SULT achieved a pTM score of 0.867 and a pLDDT score of 88.3 (Figure S3), while the best model for *Hw*SULT achieved a pTM score of 0.895 and a pLDDT score of 90.1 (Figure S4). These models were superimposed onto the structure of SULT1B1 (PDB: 3CKL), which shares the highest identity with the PAPS-binding residues of *Hw*SULT and *As*SULT. The superimposition resulted in RMSD values of 1.049 Å for *As*SULT and 1.683 Å for *Hw*SULT (Figure 6).

The *Hw*SULT and *As*SULT models revealed a remarkable degree of conservation in the overall architecture of the PAPS-binding pocket as well as in the spatial arrangement of key residues. Importantly, the histidine involved in the deprotonation of the acceptor substrate appears fully conserved, and its spatial arrangement mirrors that observed in resolved structures, suggesting that its catalytic role is likely preserved.

**Figure 7:**
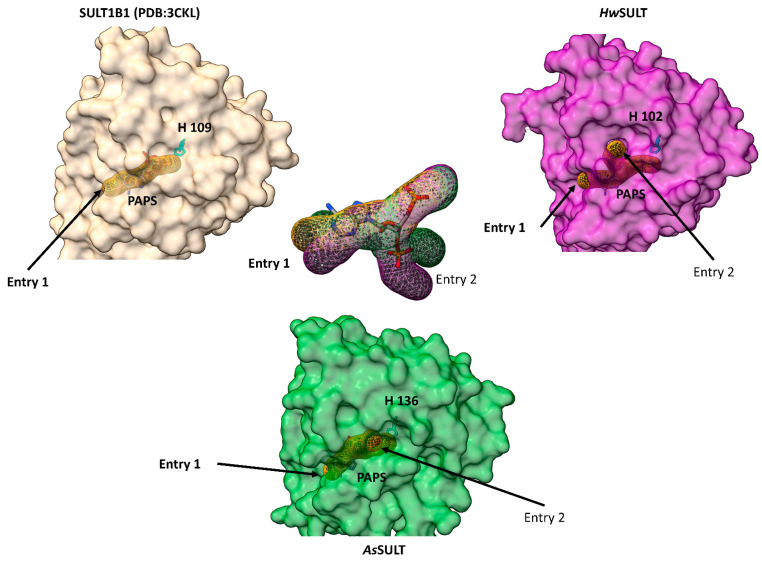
Modeling of putative entrances and tunnels leading to the PAPS-binding pocket in the SULT1B1 structure (PDB: 3CKL), *Hw*SULT, and *As*SULT models. CaveR webtool 1.2 was used to calculate entries and tunnels.

Given the apparent conservation of the PAPS-binding pocket, potential PAPS entry sites in *Hw*SULT and *As*SULT were explored. Using the Caver web tool 1.2 [53], two potential PAPS entry sites were predicted for both *Hw*SULT and *As*SULT (Figure 7), contrasting with the single-entry site identified in human SULTB1. For *Hw*SULT and *As*SULT, entry 1 is associated with a straight tunnel, while entry 2 has a curved tunnel. Both entries 1 and 2 seem to lead to the PAPS-binding pocket, but the superimposition of these tunnels reveals that entry 1 in *Hw*SULT and *As*SULT is similarly organized to that of SULT1B1, suggesting that entry 1 is more likely to be the primary entry site. Although *Hw*SULT and *As*SULT share less than 25% sequence identity with characterized SULTs, and only 23% identity with each other, it is remarkable how well the sulfate transfer mechanism may be conserved, extending from PAPS entry to the active residues involved in sulfate transfer.

### 2.3. AsSULT Shares the Same Dimerization Motif with Animal SULTs

**Figure 8:**
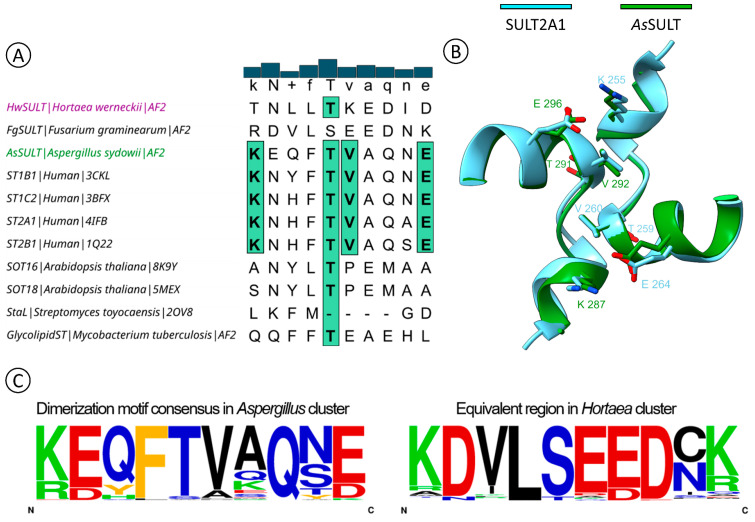
Conservation of a dimerization motif in *As*SULT and in proteins belonging to the *Aspergillus* cluster. (**A**) Putative dimerization motif (KxxxTVxxxE) in *As*SULT. Consensus threshold 70% (bolded residues). Green boxes indicate residues involved in dimerization. (**B**) *As*SULT homodimer best model overimposed on SULT2A1 homodimer. (**C**) Conservation of the dimerization motif (KxxxTVxxxE) among fungal SULTs. The frequency plot is a graphical representation of a multiple sequence alignment of the dataset of 174 sequences. The letter height is proportional to the conservation of the residue.

Many human SULTs are known to form dimeric proteins, whereas plant SULTs appear to occur as monomers [2,54]. Studies of human SULTs structures revealed a motif necessary for dimer formation, the KxxxTVxxxE motif, located at the C-terminus of the protein [55]. The MSA revealed that this motif is present within *As*SULT (Figure 8A). Moreover, almost all SULTs within the *Aspergillus* cluster possess this dimerization region (Figure 8C). Some variations occur, such as lysine replaced by arginine, threonine by serine, valine by alanine, or proline and glutamic acid by aspartic acid. However, these changes appear to be minor, as the biochemical properties of the region remain largely unchanged, suggesting that these proteins may still form dimeric structures. Conversely, the MSA revealed that the dimerization region is absent in *Hw*SULT (Figure 8A) and within the *Hortaea* cluster, with only the lysine being conserved (Figure 8C).

Consequently, attempts were made to model a homodimeric protein for *As*SULT. The best homodimeric model for *As*SULT reached a pTM score of 0.71, an ipTM score of 0.58, and a pLDDT score of 83.2 (Figure S5). AlphaFold was able to yield five coherent models for *As*SULT, each with the same interface between monomers (Figure S6). The superimposition of the *As*SULT dimer onto the structure of the human SULT2A1 resulted in an RMSD value of 2.7 Å. The dimeric *As*SULT model exhibits a protein–protein interface typical of that observed in other SULT dimers (Figure 8B). This region appears to be conserved at primary, secondary, and tertiary structure levels. Attempts to model a homodimeric structure for *Hw*SULT were unsuccessful. Five different models were produced, each with a distinct organization. In each case, the protein–protein interface showed a low degree of confidence, consistent with the absence of dimerization motif, further underscoring the structural and functional divergence between *As*SULT and *Hw*SULT.

### 2.4. Distinct Functional Profiles of HwSULT and AsSULT: A Versatile Versus Highly Specific Enzyme

**Figure 9:**
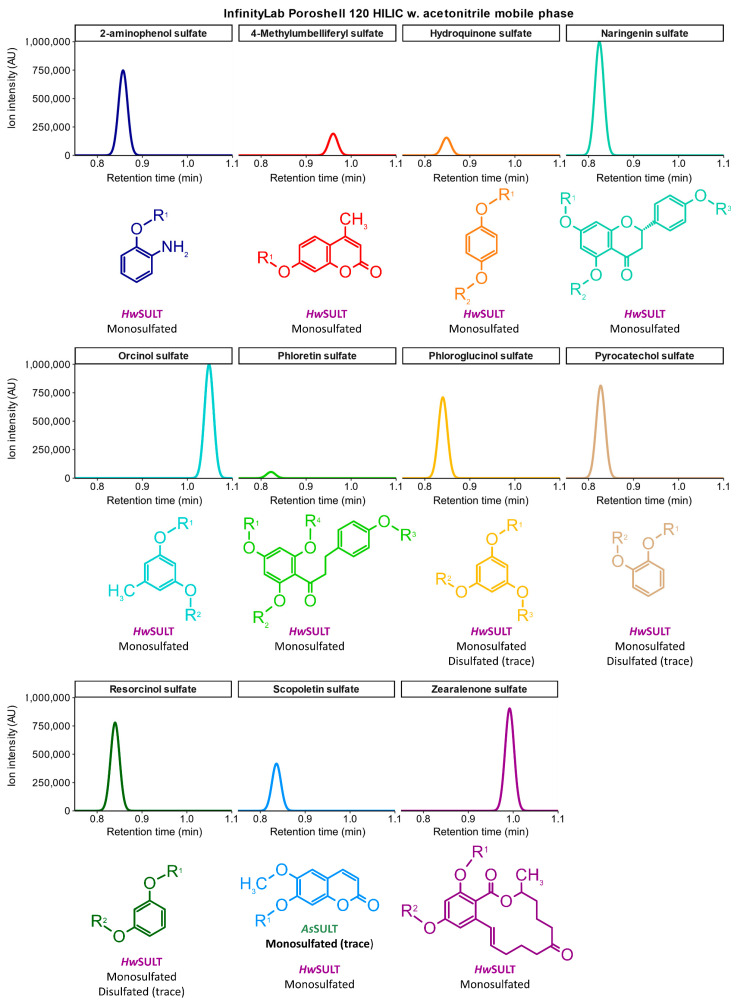
Graphical representation of substrates sulfated by *Hw*SULT and *As*SULT detected by LC-MS. R_1-2-3_ correspond to the putative sulfate position, R_1-2-3_ = H or SO_3_. The color of each chromatogram corresponds to the color of the associated chemical structure.

Phylogenetic analysis of fungal putative SULTs and structural investigations of *Hw*SULT and *As*SULT support the theory that these enzymes can use PAPS as a substrate and transfer its sulfate group onto small molecules such as secondary metabolites. Heterologous expression and biochemical characterization were conducted to further investigate these findings. *Hw*SULT was predicted to be a 36.5 kDa protein without a signal peptide, while *As*SULT was predicted to be a 35.3 kDa protein after signal peptide removal. Further structural analysis revealed a native *As*SULT predominantly existing as a homodimeric protein of approximately 70 kDa, supporting the homodimer hypothesis. In contrast, *Hw*SULT is mainly found in a monomeric form (Figure S7). The activity of recombinant SULTs was next screened using 15 different phenolic substrates of increasing size and complexity. The phylogenetic study (Figure 3) indicated that the Rv1373 SULT of *Mycobacterium tuberculosis* H37Rv^T^ may be related to *Hw*SULT. Consequently, sulfation assays were performed on various oligosaccharides, notably trehalose. Boiled enzymes and PAPS-depleted controls verified that sulfation was enzyme- and substrate-specific. Sulfated compounds were detected by HPLC-ESI-MS in negative ionization. Figure 9 and Table 1 summarize all the sulfated compounds detected after the enzymatic reaction. Experimental chromatograms and mass spectra are available in Figure S8. Biochemical screening revealed a significant contrast between *Hw*SULT and *As*SULT activities. Neither *Hw*SULT nor *As*SULT was able to sulfate oligosaccharides. *Hw*SULT demonstrated the ability to sulfate a broad range of phenolic compounds, modifying 11 out of 15 model substrates tested. In contrast, *As*SULT exhibited very low activity on the tested substrates. Three benzenediol isomers (C_6_H_6_O_2_, *m*/*z* 109.03), namely resorcinol (Benzene-1,3-diol), hydroquinone (Benzene-1,4-diol), and pyrocatechol (benzène-1,2-diol), were all effectively modified by *Hw*SULT, mainly yielding monosulfated products (C_6_H_6_O_5_S, *m*/*z* 188.9875). Trace amounts of resorcinol disulfate (C_6_H_6_O_8_S_2_, *m*/*z* 268.94, 243-fold lower than monosulfate) and pyrocatechol disulfate (C_6_H_6_O_8_S_2_, *m*/*z* 268.94, 274-fold lower than monosulfate) were observed. No sulfation was detected after exposure to *As*SULT. The presence of a methyl group, assayed with orcinol (5-methylbenzene-1,3-diol, C_7_H_8_O_2_, *m*/*z* 123.04) does not apparently affect the capacity of *Hw*SULT to sulfate this molecule (orcinol sulfate, C_7_H_8_O_5_S, *m*/*z* 203.0005). Although the substitutable positions of orcinol are the same as those of resorcinol (i.e., 1-OH and 3-OH), no orcinol disulfate was detected. A phenolic substrate containing an amine group, 2-aminophenol (C_6_H_7_NO, *m*/*z* 108.04), was sulfated by *Hw*SULT (2-aminophenol sulfate, C_6_H_7_NO_3_S, *m*/*z* 188.0031) but not by *As*SULT, suggesting that the –NH_2_ group is not suitable for *As*SULT sulfation. The number of hydroxyl groups on the benzene ring was then increased to three using phloroglucinol (benzene-1,3,5-triol, C_6_H_6_O_3_, *m*/*z* 125.02). *Hw*SULT was mainly yielded in monosulfated phloroglucinol (C_6_H_6_O_6_S, *m*/*z* 204.9827), albeit disulfated phloroglucinol (C_6_H_6_O_9_S_2_, *m*/*z* 284.9391) was observable at trace levels (134-fold lower). No trisulfated phloroglucinol was detected. Interestingly, gallic acid (3,4,5-trihydroxybenzoic acid, C_7_H_6_O_5_, *m*/*z* 169.01), which differs from phloroglucinol only by the presence of a carboxylic acid group, was not sulfated by either enzyme, suggesting that the carboxylic acid group interferes with the sulfation activity, and thus poses structural constraints on enzyme activity. Screening was extended to include more complex substrates, starting with two small mycotoxins. Patulin (C_7_H_6_O_4_, *m*/*z* 153.02) possesses a furopyran core structure containing one substitutable position located near the oxygen atom of the pyran ring. Citrinin (C_13_H_14_O_5_, *m*/*z* 249.08) is a benzopyran core molecule with one substitutable position on the benzene ring, adjacent to a carboxylic acid group. Neither molecule was sulfated by *Hw*SULT or *As*SULT. 4-methylumbelliferone (C_10_H_8_O_3_, *m*/*z* 175.03) and scopoletin (C_10_H_8_O_4_, *m*/*z* 191,037) are coumarine core molecules, with one substitutable position located on the benzene cycle. A *O-*Methyl group is adjacent to the substitutable position of scopoletin, while a methyl group is present on the pyran ring of 4-methylumbelliferone. After exposure to *Hw*SULT, scopoletin sulfate (C_10_H_8_O_7_S, *m*/*z* 270.9937) and 4-methylumbelliferyl sulfate (C_10_H_8_O_6_S, *m*/*z* 254.9971) were detected. Surprisingly, traces of scopoletin sulfate were also detected after exposure to *As*SULT. However, the ion intensity of scopoletin sulfate, obtained after exposure to *Hw*SULT, was 100-fold higher (417,000 against 4217). This latest result indicates that *As*SULT is functional but very specific for the sulfate acceptor molecule. Naringenin (C_15_H_12_O_5_, *m*/*z* 271.06) is a flavanone with three substitutable positions located on benzene moieties. Sulfated naringenin was detected (C_15_H_12_O_8_S, *m*/*z* 351.0187) after exposure to *Hw*SULT. No di- or trisulfated naringenin was detected. Phloretin (C_15_H_14_O_5_, *m*/*z* 273.08) is a chalcone with four substitutable positions located on benzene moieties. Phloretin sulfate (C_15_H_14_O_8_S, *m*/*z* 353.0358) was detected only using the *Hw*SULT enzyme. No di-, tri- or tetrasulfation was observed.

Finally, three other mycotoxins were assayed, zearalenone (C_18_H_22_O_5_, *m*/*z* 318.36), mycophenolic acid (C_17_H_20_O_6_, *m*/*z* 320.34), and ochratoxin A (C_20_H_18_ClNO_6_, *m*/*z* 403.81). Zearalenone is a complex macrocyclic molecule, with two substitutable positions located on a benzene moiety. Mycophenolic acid has a benzofuranone core structure with one substitutable position on an inner benzene moiety. The benzene ring presents a carboxymethylpentanyl, a methoxy, and methyl substitution. Ochratoxin A has a benzopyranone moiety, with an inner benzene ring presenting one chlorine atom and a substitutable position. Only zearalenone was sulfated (C_18_H_22_O_8_S, *m*/*z* 397.098) by the activity of *Hw*SULT. No sulfation occurred toward mycophenolic acid and ochratoxin A, suggesting that *Hw*SULT cannot act on an inner benzene ring and/or that chlorine might abolish enzyme activity. Thus, activity screening of *Hw*SULT reveals that this enzyme was able to sulfate eleven of the fifteen assayed substrates as long as the substitutable position was located on a benzene ring. Some substitutions, notably carboxylic acids, seem to prevent the enzyme from functioning. An inner benzene ring seems unsuitable for enzymatic activity. Only one sulfated substrate (the scopoletin sulfate) was identified, at trace levels, after the action of *As*SULT.

### 2.5. Sulfation Specifically Increased the Cytotoxicity of Zearalenone

**Figure 10:**
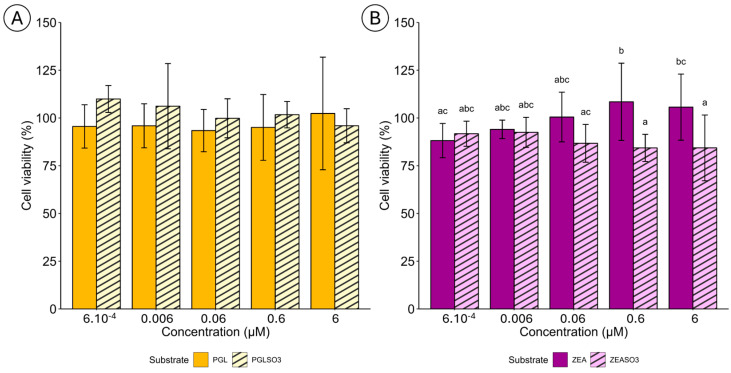
Impact of sulfation on the bioactivity of (**A**) phloroglucinol (PGL) and (**B**) zearalenone (ZEA) in feline intestinal cells (*n* = 9). One-way ANOVA results are represented by different letters, which indicate significant differences at *p* < 0.05.

In the present study, the cytotoxic effects of phloroglucinol (PGL) and zearalenone (ZEA), along with their sulfated forms (PGLSO_3_ and ZEASO_3_), were evaluated in feline intestinal cells at different concentration ranges (Figure 10).

For PGL and PGLSO_3_ (Figure 10A), no significant difference in cell viability was observed across the tested concentrations (6 × 10^−4^ µM to 6 µM). Cell viability remained around 100%, meaning neither PGL nor its sulfated form (PGLSO_3_) caused a reduction in cell viability, indicating that PGL and PGLSO_3_ are not toxic to this cell line at this concentration range. In contrast, cells exposed to ZEA (Figure 10B) exhibited a significant increase in viability at the highest concentrations, but viability decreased in the presence of the sulfated form (ZEASO_3_), with significant differences. Indeed, at higher concentrations, especially 0.6 μM and 6 μM, ZEASO_3_ significantly reduced cell viability, showing cytotoxic effects compared to the lower concentrations (6 × 10^−4^ μM and 0.006 μM), where viability remained close to 100%. Interestingly, ZEASO_3_ exhibited more cytotoxicity than its non-sulfated counterpart, as seen in the consistently lower cell viability values (which were reduced to about 75%) at the higher concentrations of 0.6 μM and 6 μM.

## 3. Discussion

Discovered over 150 years ago [1], sulfation has been the subject of extensive research. Early studies on humans revealed the critical role of sulfation, mediated by cytosolic PAPS-dependent sulfotransferases (SULTs), in numerous physiological processes. Although studies in plants have uncovered diverse PAPS-dependent sulfotransferases and sulfated secondary metabolites, their precise biological roles remain poorly understood. Several bacterial PAPS-dependent sulfotransferases have been identified [2,15,56,57,58], indicating the widespread distribution of sulfotransferases throughout the tree of life and underscoring the biological importance of sulfation in both eukaryotes and prokaryotes. Although fungi produce sulfated metabolites, the enzymes involved in such modifications remain uncharacterized, leaving fungal sulfation mechanisms largely unexplored. This work, through phylogenetic analysis, structural modeling, and biochemical characterization, represents a pioneering contribution to the elucidation of fungal sulfation mechanisms.

Data on fungal sulfotransferases are scarce. Searching the Universal Protein Resource (UniProt) database, fungal SULTs can be found only in the TrEMBL sub-database, but none are classified in the sub-database Swiss-Prot. This means that these proteins are only automatically annotated and are neither biochemically characterized nor published. In this study, through a query of the Mycocosm database containing 2612 fungal genomes, using the term PF00685, we identified 174 putative fungal SULTs that do not form a monophyletic group. These proteins are distributed across two distinct clusters (Figure 2 and Figure 3), being consistent with a previous study by Xie et al., (2020) [21]. The *Hortaea* cluster is nested within bacterial SULTs (Figure 3, cluster bootstrap 82%), while the *Aspergillus* cluster appears more closely related to eukaryotic SULTs, particularly marine taxa (e.g., brown algae). It is noteworthy that the taxonomic distribution of the fungal SULTs is restricted to the Ascomycota phylum. Early diverging fungi (e.g., Chytridiomycota, Mucoromycota) as well as Basidiomycota may lack cytosolic SULTs. However, this observation is likely due to a bias in the representation of these phyla in favor of Ascomycota, which represents up to 80% of sequenced fungal genomes (NCBI taxonomy browser consulted on 28 October 2024). On balance, only 174 proteins were identified for 1664 genomes, with some genomes containing two or three genes, possibly indicating a restricted distribution even within Ascomycota. Their limited presence in the Ascomycota phylum together with the absence of fungal monophyly could suggest that these genes were acquired after the divergence of Dikarya. This study is the first to characterize an enzyme belonging to the *Aspergillus* cluster, pointing to the potential existence of a new SULT family.

Structural analysis using sequence alignment and protein structure modeling revealed that *Hw*SULT and *As*SULT share structural features characteristic of SULTs, such as a four-stranded antiparallel beta-sheet core (Figure S9) [2], residues involved in PAPS-binding and sulfate transfer, and a conserved tunnel leading to the PAPS-binding pocket (Figure 4, Figure 6, Figure 7 and Figure 8). Historically, SULT dimerization has only been reported in animal enzymes, particularly human enzymes [2,54,59]. These homodimers are formed through interactions of a specific C-terminal region consisting of KxxxTVxxxE residues [55,60]. Interestingly, *As*SULT, as well as the *Aspergillus* cluster proteins, share this conserved region (Figure 8). Conversely, *Hw*SULT and proteins of the *Hortaea* cluster lack this feature (Figure 8). The characterization of human SULTs could suggest that dimerization influences protein stability and regulation [61,62], while the disruption of this motif does not appear to abolish the activity of the monomeric form [63,64]. Noteworthy, the PAPS-dependent bacterial SULT StaL from *Streptomyces toyocaensis* apparently exists as a dimer without the KxxxTVxxxE dimerization motif, which suggests that the latter may not be universal among SULTs [50].

To gain deeper insights into the evolutionary and functional implications of this phylogenetic separation among fungal SULTs, a representative enzyme from a marine fungus of each cluster was biochemically characterized. For this purpose, the *Hw*SULT from *Hortaea werneckii* UBOCC-A-208029 and the *As*SULT from *Aspergillus sydowii* UBOCC-A-108050 were used. Both recombinant *As*SULT and *Hw*SULT were overproduced in *E. coli* and analyzed by calibrated size exclusion chromatography. *As*SULT appears to exist in a homodimeric form, consistent with dimerization predictions, while *Hw*SULT appears to be a monomeric enzyme. This dichotomy between the *Aspergillus* and *Hortaea* clusters raises questions about the potential role of dimerization among fungal SULTs and is reminiscent of that existing between animal SULTs, which are mostly dimeric, and plant SULTs, which are predominantly monomeric. Further analysis of *As*SULT, such as disruption of the KxxxTVxxxE motif, together with extended biochemical characterization, could help explain the role of dimerization in fungi. Thus, the dimerization of SULTs is not limited to animal SULTs and could be more widespread among eukaryotes, like fungi.

To confirm the enzymatic function of these two SULTs, i.e., to transfer a sulfate group from PAPS to an acceptor molecule (a secondary metabolite-type molecule), the recombinant proteins were overproduced and biochemically characterized. The activity screening revealed that both recombinant enzymes were active, confirming their ability to use PAPS, as suggested by the structural analysis. Each enzyme displayed distinct functional profiles.

The identification of the *Aspergillus* cluster and the *As*SULT characterization constitute the earliest evidence for the existence of a new fungal SULT family. Only scopoletin, a plant coumarin [65], was sulfated by *As*SULT, suggesting a significant specificity. However, sulfated scopoletin was only detected at trace levels, which could indicate that this substrate is chemically distant from the natural substrate. Numerous specialized metabolites have been identified in *A. sydowii*, including chromans and coumarins [66]. Thus, an investigation of secondary metabolites forming a Biosynthetic Gene Cluster (BGC) was conducted using AntiSMASH v.7 fungal version (FungiSMASH) on the *A. sydowii* CBS 593.65 genome [67]. However, no results demonstrated that *As*SULT could be part of a known or characteristic specialized metabolite BGC (e.g., polyketides, non-ribosomal peptides, terpenes) [68]. These results suggest that *As*SULT is unlikely to sulfate a known specialized metabolite. The genomic environment of *As*SULT was therefore examined using InterProScan and could be evocative of the biosynthesis of an unknown molecule (Figure S12). *As*SULT is notably flanked by a gene that could encode a fungal “regulator of secondary metabolite biosynthesis”. This putative BGC also contains genes that could encode for hydrolase (IPR052897), metalloproteinase (IPR053002), phosphomevalonate kinase (IPR005919), and FAD-linked oxidoreductase (IPR050416). In light of these findings, *As*SULT and its surrounding genes could be associated with the biosynthesis of a specific molecule, which could be unrelated to secondary metabolism, as suggested by the results of AntiSMASH v.7 fungal version. Further characterization of this putative BGC along with identification of sulfated molecules in vivo could provide answers to this question. Despite three phylogenetic studies (Xie et al., (2020) [21], Figure 2 and Figure 3), the phylogenetic placement of the *Aspergillus* cluster is uncertain. However, the three studies seem to converge on the conclusion of an evolutionary relationship closer to eukaryotes (animals, plants, algae) than prokaryotes. Based on current knowledge, the presence of a conserved and visibly functional dimerization motif could suggest that the *Aspergillus* cluster is closer to animal clusters. Future studies on marine eukaryotes, such as brown algae, might provide new perspectives.

Phylogenetically, fungal SULTs are not monophyletic, which could reflect differences in structures, substrates, biochemical activities, and biological functions. Although *As*SULT and *Hw*SULT share characteristics typical of PAPS-dependent SULTs, their structures differ considerably (Figures S1 and S9). Unlike *As*SULT, the genomic environment associated with the *Hw*SULT gene does not support any hypotheses about its biological function (Figure S13). Biochemically, *Hw*SULT exhibited broad substrate specificity, sulfating various substrates ranging from a small phenolic molecule (e.g., phloroglucinol) to larger macrolides (e.g., zearalenone). Substrate limitations were observed (Figure S10) for heterocyclic compounds (e.g., the pyran ring of patulin), suggesting that *Hw*SULT is only able to sulfate phenolic moieties. The presence of some substitutions, such as carboxylic acid (e.g., gallic acid, citrinin), appears to abolish *Hw*SULT activity. This observation could suggest that these substitutions may prevent the substrate from accessing or being correctly oriented within the catalytic site, potentially due to steric bulk or charge interferences. *Hw*SULT did not sulfate molecules such as mycophenolic acid or ochratoxin A. For these two molecules, the substitutable position is not located at a terminal position (Figure S10). Thus, access to the catalytic site would be restricted to the terminal positions of acceptor substrates, indicating that *Hw*SULT would be an exo-sulfotransferase and explaining the wide range of molecules that can be sulfated by the enzyme. *Fg*SULT1, studied by Xie et al., (2020) [21], belongs to the same cluster as *Hw*SULT (Figure 2, 49% sequence identity between *Fg*SULT1 and *Hw*SULT). These authors demonstrated the ability of this enzyme to transfer a sulfate group on benzenediol lactones, a class of polyketide widely produced in fungi [69]. Apart from a 2,4-dihydroxybenzaldehyde motif, which appears to be required for *Fg*SULT1 activity, no other conditions were identified. *Hw*SULT and *Fg*SULT1 exhibit similar characteristics, including a broad range of acceptor substrates and relatively low specificity. However, *Hw*SULT seems to be active toward a wider spectrum of substrates than *Fg*SULT1, in particular simple phenolic compounds, flavonoids, and hydroxycoumarins. By sulfating these molecules, *Hw*SULT does not appear to be constrained by a 2,4-dihydroxybenzaldehyde motif, which has been proposed as a requirement for *Fg*SULT1 activity [21]. *Fg*SULT1 was active toward lasilarin, a dihydroxyphenylacetic acid lactone (DAL), but was inactive toward zearalenone, which is a resorcylic acid lactone (RAL) (Figure S11) [21,70], despite both DAL and RAL molecules possessing a 1,3-benzenediol moiety. In our study, *Hw*SULT was able to sulfate zearalenone. However, no DALs were assayed. Given the shared evolutionary history of *Hw*SULT and *Fg*SULT1, future assays on various RALs and DALs could help clarify the differences and similarities between the two enzymes.

The global phylogenetic analysis (Figure 3) seems to point to a potential evolutionary relationship between the *Hortaea* cluster and bacterial enzymes (bootstrap value 82%). However, this cluster is heterogeneous and contains a broad taxonomic diversity of bacteria. No association clearly appears between a specific bacterial group and these fungal groups. Regarding bacteria, a few bacterial SULTs involved in the biosynthesis of specialized metabolites have also been identified, notably in *Streptomyces* species [56,71,72,73,74]. Notable characterizations include StaL from *Streptomyces toyocaensis* [50,56], which is involved in the biosynthesis of the glycopeptide-aglycone A47934, Cpz8 from *Streptomyces* sp. MK730–62F2 [71], which is involved in caprazamycin biosynthesis, and ToTs from *S. lividans* TK64 [74], which is involved in the biosynthesis of Totopotensamide C. It is noteworthy that Cpz8 was able to sulfate various phenolic substrates, including 4-methylumbelliferone, like *Hw*SULT. Interestingly, glycopeptide sulfation by *Streptomyces* species could be a key factor in preventing antibiotic resistance development in other organisms [75], suggesting a role for specialized metabolite sulfation. Other examples of bacterial SULTs include a SULT from *Lyngbya majuscule*, a marine cyanobacteria, which is required for the biosynthesis of Curacin A [76], and the *Bt*SULT from *Bacteroides thetaiotaomicron* (*Bacteroidota*), a human gut bacterium, which is involved in the sulfation of dietary steroids such as cholesterol [77]. Additionally, several *Mycobacterium* (*Actinomycetota*) possess SULTs [78], which are involved in the sulfation of membrane glycolipids such as the Rv1373 SULT from *Mycobacterium tuberculosis* H37Rv^T^, which is the only characterized SULT in the *Hortaea*/bacteria cluster [51,79]. However, Rv1373 SULT activity is fundamentally different from that of *Hw*SULT and *Fg*SULT1. While these fungal SULTs are involved in the sulfation of small phenolic molecules, Rv1373 SULT sulfates the oligosaccharide moiety of membrane glycolipids [51,79]. To echo the apparent phylogenetic relationship between these two enzymes, attempts were made to sulfate various oligosaccharides, including trehalose; however, no sulfation occurred. This major functional difference raises questions about its presence in the same phylogenetic cluster as the fungal enzymes, as well as the function of the other bacterial enzymes present in this cluster. Therefore, although it remains to be elucidated, there could be a relationship between the *Hortaea* cluster and bacterial enzymes. Genome mining and targeted phylogenetic analysis of SULT relationships between bacteria and fungi, together with biochemical characterization, could help to elucidate this link.

Sulfation is known to significantly modify the chemical properties of various compounds, leading to the acquisition, enhancement, or loss of activity [2]. *Hw*SULT demonstrates the ability to sulfate both fungal and non-fungal molecules, notably zearalenone, a resorcylic acid lactone produced by *Fusarium* species. This mycotoxin possesses estrogenic activities through its binding to estrogen receptors of animals. Considering the public health concerns associated with zearalenone (ZEA), understanding how sulfation could affect its toxic properties appears valuable. Absorption through the intestine, which serves as the first physical barrier to foreign substances, is crucial to understanding ZEA’s bioactivity. As mycotoxin-contaminated feed is absorbed, the intestine and its epithelial cell layer will be exposed to high concentrations of the toxin, which will undoubtedly affect intestinal health. Thus, the effect of sulfation on the bioactivity of ZEA on feline intestinal cells close to non-tumoral human intestinal cells was examined. No cytotoxicity was observed for non-sulfated ZEA over the entire concentration range assayed. Rather, a slight increase in mitochondrial activity was observed at the highest concentrations (0.6 and 6 µM), suggesting increased cellular proliferation. These results are in agreement with previous studies on Intestinal Porcine Epithelial Cells (IPEC-1 from jejunum and ileum and IPEC-J2 from jejunum), where no cytotoxicity or even increased mitochondrial activity was observed for these concentration ranges [80,81]. Interestingly, these authors observed a decrease in IPEC viability above 10 µM. This could suggest that a cytotoxic effect could have been observed on feline intestinal cells at higher concentrations. After sulfation using *Hw*SULT, the activity of ZEA was modified. Significant cytotoxicity as low as 0.6 µM was observed with ZEASO_3_, where non-sulfated ZEA previously showed a proliferative effect. Assuming a similar response between IPECs and feline intestinal cells, the concentration of ZEASO_3_ required to achieve a cytotoxic response would be approximately 20-fold lower than when in its non-sulfated form. On the other hand, previous studies on tumoral human cell lines, such as HepG2 (human liver cancer cells), Caco-2 (colorectal adenocarcinoma cells), and THP-1 (monocytes) [82,83], demonstrated ZEA’s dose-dependent cytotoxicity. These different effects may be due to the loss of some endocrine receptors. Phloroglucinol and its derivatives are produced by many organisms, in particular plants, algae, and microorganisms. These compounds have a wide range of properties, which makes them pharmacologically attractive [84]. After sulfation by *Hw*SULT, both phloroglucinol (PGL) and sulfated phloroglucinol (PGLSO_3_) were investigated for their activity on feline intestinal cells. Neither PGL nor PGLSO_3_ exhibit cytotoxic activity against feline intestinal cells. Thus, at the concentrations tested and for the model used, sulfation has no observable impact on cytotoxicity. However, PGL is also known as an anti-inflammatory or antioxidant molecule [85]. Complementary experiments evaluating how sulfation might alter these bioactivities could be of great interest to potential reveal additional pharmacological implications. These results show how sulfation can potentiate the bioactivity of certain molecules and raise questions about the ecological and physiological reasons behind why fungi may benefit from sulfating such molecules. This comparison suggests that sulfation may have implications on the metabolization and toxicity of such compounds in biological systems. Overall, these results suggest that cytotoxicity seems to be molecule-dependent, as PGL, in both its normal and sulfated forms, exhibited minimal effects on cell viability, even at high concentrations. In contrast, ZEA, an estrogenic mycotoxin and an endocrine disrupting compound, showed no dose-dependent cytotoxicity effect. ZEA is also characterized by a non-monotonic dose–response curve [86]. In contrast, ZEASO_3_ appears to be more toxic than ZEA, particularly at higher concentrations, indicating that sulfation may enhance some of the harmful effects of ZEA on cell viability. Further experiments would be very interesting to demonstrate any loss of endocrine disrupting properties after sulfation, particularly on healthy cells.

## 4. Materials and Methods

### 4.1. Fungal Strains

The two fungal strains used in this study were isolated from a hydrothermal vent ecosystem in the Mid-Atlantic Ridge (Rainbow site, 2300 m deep) during the MoMARDREAM oceanographic campaign [87]. *Hortaea werneckii* Mo34 (UBOCC-A-208029) was isolated from the mussel *Bathymodiolus azoricus* [41], and *Aspergillus sydowii* Mo4 (UBOCC-A-108050) was isolated from the shrimp *Rimicaris exoculata* [40]. Both strains were grown on Potato Dextrose Agar medium supplemented with 3% sea salt (PDA SS) (Sigma).

### 4.2. Bacterial Strains

*Escherichia coli* K-12 JM109 competent cells [*end*A1, recA1, *gyr*A96, *thi*, *hsd*R17 (r_K_^−^, m_K_^+^), *rel*A1, *sup*E44, ∆(*lac-pro*AB), [F’ *tra*D36, *pro*AB, *lac*I^q^ZΔM15]] was used as the host strain for the validation of SULT gene sequences. *E. coli* BL21-CodonPlus (DE3)-RIPL [*E. coli* B F^−^ *omp*T *hsd*S (r_B_^−^, m_B_^−^) *dmc*^+^ *Tet*^r^ galλ(DE3) *end*A Hte [*arg*U *pro*L *Cam*^r^] [*arg*U *ile*Y *leu*W Strep/Spec^r^]] was used to produce recombinant proteins. Bacterial strains were grown using Lysogeny Broth (LB) (10 g·L^−1^ peptones, 10 g·L^−1^ yeast extract, and 10 g·L^−1^ NaCl).

### 4.3. Cell Culture Conditions

Feline intestinal epithelial cells were obtained following the protocol described by Desmarets 2013. Cells were transported in ice-cold Dulbecco’s Modified Eagle Medium (DMEM; Gibco BRL, Merelbeke, Belgium) and cultured in DMEM/F-12 supplemented with 100 U·mL^−1^ penicillin, 0.1 mg·mL^−1^ penicillin/streptomycin, 10% FBS (Gibco BRL, Waltham, MA, USA), 10 ng·mL^−1^ epidermal growth factor (Sigma-Aldrich, St. Louis, MO, USA), 1% insulin–transferrin–selenium–X (Invitrogen, Carlsbad, CA, USA), 100 nM hydrocortisone (Sigma-Aldrich, St. Louis, MO, USA), and 1% non-essential amino acids 100× (Gibco BRL, Waltham, MA, USA). Cultivation was performed at 37 °C in a 5% CO_2_ atmosphere. Culture medium was replaced every 2–3 days, and cell morphology was monitored daily by light microscopy (Olympus Corporation, Tokyo, Japan). Once the cells reached 80% confluence, they were seeded in 96-well plates. This study was approved by the Local Ethical and Animal Welfare Committee of the Faculty of Veterinary Medicine of Ghent University (EC2012/042).

### 4.4. Chemicals

The 3′-Phosphoadenosine 5′-phosphosulfate (PAPS), phloroglucinol, hydroquinone, pyrocatechol, resorcinol, orcinol, 2-aminophenol, 4-methylumbelliferone, gallic acid, scopoletin, phloretin, naringenin, patulin, citrinin, mycophenolic acid, ochratoxin A, zearalenone, glucose, galactose, cellobiose, lactose, maltose, maltotriose, and trehalose were purchased from Sigma-Aldrich (St. Louis, MO, USA). *N*-acetyl lactosamine and raffinose were purchased from Biosynth (Staad, Switzerland).

### 4.5. Bioinformatic Analysis

#### 4.5.1. Gene Identification, Dataset Building, and Analysis

Putative SULT genes were identified from fungal genomes using the Pfam ID PF00685 (Sulfotransfer_1) as a query. All matching sequences were extracted in FASTA format and analyzed using Geneious Prime 2024.0.5. The global set was analyzed using InterproScan version 5.72-103.0 [42] to confirm the initial Pfam-based predictions. Sequences lacking PF00685 or PTHR11783, or those shorter than 120 amino acids or missing PAPS-binding motifs, were removed from the dataset. Transmembrane domains and signal peptides were predicted using DeepTMHMM [88] and SignalP v.6 [89], respectively. Frequency plots were created using WebLogo 2.8.2 [90].

#### 4.5.2. Homology Model Building

*Hw*SULT and *As*SULT homology models were built using AlphaFold 2 [45]. The best models were aligned with the human SULT1B1 crystal structure (PDB: 3CKL) using USCF ChimeraX v1.8 [91]. CaveR webtool v 1.2 [53] was used to analyze putative pocket and tunnel volume.

### 4.6. Global Phylogenetic Tree of PAPS-Dependent Sulfotransferases and Phylogeny of Sulfotransferases from Fungi

For the global phylogeny of PAPS-dependent sulfotransferases, 2010 eukaryotic and prokaryotic sulfotransferases sequences were aligned using MAFFT v.7 [92] through the L-INS-i algorithm and the mafft—add function. Alignments were visualized in Jalview software v.11.0 [93], and poorly aligned regions were removed, leading to 160 positions for the phylogenetic analysis. The dataset of fungal PAPS-dependent sulfotransferases, including 174 fungal sulfotransferases, along with 11 human SULTs and 16 *A. thaliana* SOTs, was established. The sulfotransfer_1 domains were extracted from sequences and aligned using MAFFT v.7 [92] through the E-INS-I algorithm. Phylogenetic trees were built using the maximum likelihood method in RAxML v. 8.2.4 [94] with the LG substitution matrix as an evolutionary model [95]. Tree reliability was tested using 100 bootstrap replicates [96]. The global phylogenetic tree and deduced subtrees were generated using MEGA v.6 [97], while the tree of fungi was built using Mega v.11 [98]. Both trees were rooted on the midpoint.

### 4.7. Cloning of H. werneckii UBOCC-A-208029 and A. sydowii UBOCC-A-108050 SULT Genes

Both *Hw*SULT and *As*SULT genes were cloned from mRNA extracted using the Nucleospin^®^ RNA plant and Fungi kit (Macherey-Nagel, Hoerdt, France). cDNAs were synthesized through reverse transcription using the ThermoScript RT-PCR System and Oligo(dT)_20_ primers. SULT genes were subsequently synthesized in a 50 µL PCR reaction containing Phusion^®^ HF DNA Polymerase (New England Biolabs, Ipswich, MA, USA), 1 X Phusion Buffer, 0.2 mM dNTPs, 3% of DMSO, 0.5 µM of each primer (*Hw*SULT-F: 5′-GAGATCGGATCCATGGACTCCGTAGTTGGATCAC, *BamH*I restriction site underlined, *Hw*SULT-R: 5′-GAGATCGGTACCCTACCGACCCTTTGCAGCG, *Kpn*I restriction site underlined, *As*SULT-F: 5′-CACCATCACCATCACGGATCCACCAAGCTTCGAGTCTTCGAA *BamH*I, restriction site underlined, and *As*SULT-R: 5′-CTGTCGTTTGACGGATTGTGACTGCAGCCAAGCTTAATTAG, *Pst*I restriction site underlined), and 50 ng of cDNA. The amplification protocol was as follows: initial denaturation at 98 °C for 5 min, followed by 40 cycles of 1 min at 98 °C, 1 min at 54 °C, and 2 min at 72 °C, with a final elongation step at 72 °C for 10 min. The resulting *Hw*SULT and *As*SULT cDNA products were purified using 0.8% agarose gel and the Nucleospin^®^ Gel and PCR Clean-up kit (Macherey-Nagel, Hoerdt, France), and then cloned into pQE-80L plasmid following digestion with *Kpn*I/*Pst*I or *BamH*I/*Kpn*I and ligated using T4 DNA Ligase (Promega, Madison, WI, USA). *E. coli* JM109 was transformed with the recombinant plasmids, and individual clones were sequenced for validation by Eurofins Genomics (Cologne, Germany).

### 4.8. Protein Expression and Purification

*E. coli* BL21-CodonPlus (DE3)-RIPL cells were transformed with pQE-80L-*Hw*SULT or pQE-80L-*As*SULT. The transformed *E. coli* cells were grown in LB containing 100 µg·mL^−1^ ampicillin at 37 °C until the OD_600nm_ reached 0.6–0.8. Protein expression was induced with 1 mM Isopropyl-*β*-D-thiogalactopyranoside (IPTG), followed by 21 h of incubation at 20 °C. Cells were centrifuged at 6000× *g* for 15 min at 4 °C, and the pellets were resuspended in 50 mM Tris-HCl, 500 mM NaCl, 20 mM Imidazole, pH 7.5 buffer, supplemented with lysozyme. Cells were disrupted by sonication (3 cycles of 30 s with 1 min rest on ice between each cycle, power 40%), and cellular debris was removed by centrifugation at 20,000× *g* for 1 h at 4 °C. His-tagged proteins were purified at room temperature, on a Nickel (Ni) tetradentate absorbent (NTA) column HisTrap FF (GE Healthcare, Chicago, IL, USA), using an Äkta start system (GE Healthcare, Chicago, IL, USA) equipped with a UV detector set to 280 nm. Elution was conducted using a linear gradient from 0% to 100% of buffer B (50 mM Tris-HCl, 500 mM NaCl, 500 mM Imidazole, pH 7.5), and 1.5 mL fractions were collected during elution. Protein purification was enhanced using a Sephacryl S-200 (GE Healthcare, Chicago, IL, USA) with 50 mM Tris-HCl, 200 mM NaCl, pH 7.5 buffer. Fractions of 2 mL were collected. Protein size and purity were analyzed using SDS-PAGE (12% polyacrylamide). Fractions containing the protein of interest were pooled, dialyzed, and concentrated using Vivaspin 20^®^ Ultrafiltration Unit 30 kDa (Sartorius, Göttingen, Germany).

### 4.9. Sulfation Essays

Sulfation assays were performed in 50 µL of mixture containing 50 mM Tris-HCl (pH 7), 10 mM MgCl_2_, 10 mM NaF, 2 mM PAPS, specific substrate (Table 2), and 20 µg of *Hw*SULT or *As*SULT. Sulfation assays were also conducted on glucose, galactose, cellobiose, lactose, maltose, maltotriose, trehalose, *N*-acetyl lactosamine, and raffinose at 1 mg·mL^−1^. Assays were performed for 2 h at approximately 20 °C. Controls were conducted using boiled enzymes or PAPS-depleted reactions.

After the enzymatic reaction, three volumes of acetonitrile were added, and the samples were filtered through 0.45 µm filters. Sulfated products were analyzed using an Agilent 6530 Accurate-Mass Q-ToF LC-MS (Agilent Technologies, Santa Clara, CA, USA) equipped with an InfinityLab Poroshell 120 HILIC column (2.1 × 100 mm, 2.7 µm) (Agilent technologies, Santa Clara, CA, USA) to separate products. Isocratic elution was performed with 10% of 10 mM ammonium formate buffer (containing 0.1% formic acid) and 90% of acetonitrile (containing 0.1% formic acid) at 0.25 mL·min^−1^.

### 4.10. Secondary Metabolite Extraction

*H. werneckii* UBOCC-A-208029 and *A. sydowii* UBOCC-A-108050 were grown on PDA SS for 14 days. Fungal biomass and culture medium were harvested and homogenized with four volumes of acetonitrile (ACN) or ethyl acetate (EtAc), followed by sonication for 15 min and agitation for an additional 15 min. The mixtures were centrifuged at 6000 rpm for 15 min at 4 °C, and the supernatants were collected and evaporated to dryness. Dry extracts were resuspended with 200 µL of ACN or EtAc.

Extracts were analyzed using Agilent 6530 Accurate-Mass Q-ToF LC-MS (Agilent Technologies, Santa Clara, CA, USA). Molecules were separated on a Zorbax Extend-C18 column (2.1 × 50 mm, 1.8 μm) (Agilent technologies, Santa Clara, CA, USA) using two gradient elution methods: (i) ammonium formate buffer (containing 0.1% formic acid) and ACN (containing 0.1% formic acid), with a gradient from 5% to 100% ACN over 32 min at 0.2 mL·min^−1^, and (ii) water (with 0.1% formic acid and 0.5% acetic acid) and methanol (containing 0.1% formic acid and 0.5% acetic acid), with a gradient from 10 to 100% methanol over 20 min at 0.3 mL·min^−1^.

### 4.11. Cytotoxicity Evaluation by Mitochondrial Activity

Feline intestinal cells were plated in 96-well tissue culture plates at a density of 3 × 10^4^ cells/well. The mitochondrial activity of cells exposed to phloroglucinol, zearalenone, and their sulfated forms was evaluated using the MTS assay. After 48 h of exposure (acute exposure), cells were washed with PBS, followed by an incubation with 20 μL of freshly prepared MTS/PMS for 3 h. The amount of soluble formazan (MTS metabolite) was then quantified by measuring the absorbance at 490 nm using a Multiskan FC plate reader (Thermo Scientific, Madison, WI, USA). Cell viability obtained for the negative control (cell cultures not exposed to molecules) was defined as 100% and affected fraction as 0%. Affected fraction means that the percentages of three independent experiments ± standard deviation of the mean (SD) were used for statistical analyses.

### 4.12. Statistical Analysis

Statistical analyses were performed using R v 4.1.1 and the Rstudio “Chocolate Cosmos” release. Normality and homoscedasticity were verified using the Shapiro–Wilk test and Levene test, respectively. One-sample *t*-tests were used to compare cell viability to a reference value of 100%. The effect of sulfation and concentration on cell viability were evaluated using one-way ANOVA followed by a post hoc Tukey test.

## 5. Conclusions

In this study, through an integrated approach involving phylogenetic analyses, protein modeling, heterologous expression, and biochemical characterization, we identified and characterized the first two SULTs from marine fungi: *Hw*SULT from *Hortaea werneckii* UBOCC-A-208029 and *As*SULT from *Aspergillus sydowii* UBOCC-A-108050, both isolated from deep-sea hydrothermal vents and belonging to the aryl sulfotransferase family. Additionally, 174 fungal SULTs within the Ascomycota phylum were identified and classified within the aryl sulfotransferase family, considerably expanding the known repertoire of fungal sulfotransferases. Fungal SULTs do not form a monophyletic group and are divided into two distinct clusters. The two characterized SULTs, representative of each group, are structurally (notably at the quaternary structure level) and functionally dissimilar. *As*SULT, a homodimeric protein that appears to have high substrate specificity, shares 25% sequence identity with *Hw*SULT, a monomeric enzyme with a high activity toward a broad range of phenolic substrates. The genomic environment and biochemical characterization suggest that *As*SULT could play a role in a biosynthesis pathway of an unknown molecule, whereas the biological function of *Hw*SULT remains undefined. Further studies are needed to clarify the biological roles of both enzymes. Notably, the identification and characterization of sulfated molecules in these fungal strains could help validate these hypotheses and lead to the identification of new sulfated bioactive compounds, molecules that are still rare in fungi.

## Figures and Tables

**Table 1:** Substrates and sulfated products detected after enzymatic reactions. Formulas were provided by MassHunter Qualitative Analysis version B.06.00 based on the isotopic profile of each detected molecule.

Initial Substrate				Sulfated Molecule			
Substrate	Formula	RT (min)	*m*/*z*	Formula	RT (min)	*m*/*z*	Enzyme
Resorcinol	C_6_H_6_O_2_	0.83	109.03	C_6_H_6_O_5_S	0.84	188.9875	*Hw*SULT
				C_6_H_6_O_8_S_2_	0.84	268.94	*Hw*SULT
Pyrocatechol	C_6_H_6_O_2_	0.82	109.03	C_6_H_6_O_5_S	0.83	188.9875	*Hw*SULT
				C_6_H_6_O_8_S_2_	0.83	268.94	*Hw*SULT
Hydroquinone	C_6_H_6_O_2_	0.84	109.03	C_6_H_6_O_5_S	0.84	188.9875	*Hw*SULT
Orcinol	C_7_H_8_O_2_	1.00	123.04	C_7_H_8_O_5_S	1.05	203.0005	*Hw*SULT
2-aminophenol	C_6_H_7_NO	0.86	108.04	C_6_H_7_NO_3_S	0.86	188.0031	*Hw*SULT
Phloroglucinol	C_6_H_6_O_3_	1.1	125.02	C_6_H_6_O_6_S	0.84	204.9827	*Hw*SULT
				C_6_H_6_O_9_S_2_	0.84	284.9391	*Hw*SULT
Gallic acid	C_7_H_6_O_5_	1.15	169.01	/	/	/	/
Patulin	C_7_H_6_O_4_	1.16	153.02	/	/	/	/
Citrinin	C_13_H_14_O_5_	1.10	249.08	/	/	/	/
4-methylumbelliferone *	C_10_H_8_O_3_	-	175.03	C_10_H_8_O_6_S	0.96	254.9971	*Hw*SULT
Scopoletin	C_10_H_8_O_4_	1.3	191.037	C_10_H_8_O_7_S	0.84	270.9937	*Hw*SULT/*As*SULT
Naringenin	C_15_H_12_O_5_	1.08	271.06	C_15_H_12_O_8_S	0.82	351.0187	*Hw*SULT
Phloretin	C_15_H_14_O_5_	1.08	273.08	C_15_H_14_O_8_S	0.82	353.0358	*Hw*SULT
Zearalenone	C_18_H_22_O_5_	1.10	318.36	C_18_H_22_O_8_S	0.99	397.098	*Hw*SULT
Mycophenolic acid	C_17_H_20_O_6_	1.14	320.34	/	/	/	/
Ochratoxin A	C_20_H_18_ClNO_6_	1.13	403.81	/	/	/	/

* Non-sulfated 4-methylumbelliferone was not detected using the method described in Materials and Methods, 4.9. However, it was detected using the method described in 4.10.

**Table 2:** Substrates used for sulfation assays.

Substrate	Concentration (mM)
Resorcinol	45.4
Pyrocatechol	45.4
Hydroquinone	45.4
Orcinol	80.6
2-aminophenol	45.8
Phloroglucinol	0.6
Gallic acid	29.4
Patulin	6.5
Citrinin	0.8
4-methylumbelliferone	0.2
Scopoletin	26
Naringenin	18.3
Phloretin	18.2
Zearalenone	0.628
Mycophenolic acid	1.12
Ochratoxin A	0.1

## Data Availability

The data presented in this study are available on request from the corresponding author.

## Supplementary Materials

The following supporting information can be downloaded at: https://www.mdpi.com/article/10.3390/md22120572/s1. Figure S1: percent identity matrix of multiple sequence alignment between *Hortaea werneckii UBOCC-A-*208029 putative SULT, *Fusarium graminearum* PH-1 *Fg*SULT1, *Aspergillus sydowii* UBOCC-A-108050 putative SULT, Human SULT1B1, SULT1C2, SULT2A1, and SULT2B1 (PDB accession number: 3CKL, 3BFX, 4IFB, 1Q22), *Arabidopsis thaliana* SOT16 and SOT18 (PDB accession number: 8K9Y, 5MEX), *Streptomyces toyocaensis* Stal (PDB accession number: 2OV8), and *Mycobacterium tuberculosis* glycolipidST; Figure S2: percent identity matrix of identified PAPS-binding residues between *Hortaea werneckii* UBOCC-A-208029 putative SULT, *Aspergillus sydowii* UBOCC-A- 108050 putative SULT, Human SULT1B1, SULT1C2, SULT2A1, and SULT2B1 (PDB accession number: 3CKL, 3BFX, 4IFB, 1Q22), Arabidopsis thaliana SOT16 and SOT18 (PDB accession number: 8K9Y, 5MEX), Streptomyces toyocaensis Stal (PDB accession number: 2OV8), and Mycobacterium tuberculosis glycolipidST; Figure S3: (A) analysis of sequence coverage of *As*SULT per position. (B) analysis of the quality confidence of the five *As*SULT models, pLDDT per position. (C) Plots of the predicted alignment error (PAE) identified per *As*SULT model; Figure S4: (A) analysis of sequence coverage of *Hws*SULT per position. (B) Analysis of the quality confidence of the five *Hw*SULT models, pLDDT per position. (C) Plots of the predicted alignment error (PAE) identified per *Hw*SULT model; Figure S5: (A) analysis of sequence coverage of *As*SULT homodimer per position. (B) Analysis of the quality confidence of the five *As*SULT homodimer models, pLDDT per position. (C) Plots of the predicted alignment error (PAE) identified per *As*SULT homodimer model. Figure S6: dimer interface of the *As*SULT homodimer, colored by confidence. A to E correspond to rank 1 to rank 5 model; Figure S7: size exclusion chromatogram of *As*SULT and *Hw*SULT with associated 12% SDS-PAGE analysis; Figure S8: experimental chromatogram and mass spectra of each molecule sulfated by *Hw*SULT (purple) or *As*SULT (green); Figure S9: secondary structure highlights of *Hw*SULT, *As*SULT, and SULT1B1 (PDB: 3CKL); Figure S10: chemical structures of substrates not sulfated by either *Hw*SULT or *As*SULT; Figure S11: chemical structures of Lasilarin (Xie et al., 2020) [21] and Zearalenone; Figure S12: organization of genes surrounding *As*SULT. Each arrow represents a gene with its corresponding annotation according to the InterPro analysis; Figure S13: organization of genes surrounding *Hw*SULT. Each arrow represents a gene with its corresponding annotation according to the InterPro analysis; Table S1: accession number, organismal origin, and Source DataBank of proteins used to build the phylogenetic tree of putative fungal SULTs; Table S2: accession number, organismal origin, and Source DataBank of proteins used to build the phylogenetic tree of St1 SULT family (PF00685); Table S3: PAPS-binding residues. Bold residues were extracted from the three-dimensional structures (PDB). Non-bold residues were inferred from the multiple sequence alignment.
