# Supplementary material for: Identification and Characterization of Two Aryl Sulfotransferases from Deep-Sea Marine Fungi and Their Implications in the Sulfation of Secondary Metabolites
Source: Mar Drugs. 2024 Dec 20;22(12):572. doi: 10.3390/md22120572 (PMC11677658; doi:10.3390/md22120572)
Supplement: Supplementary file 1 [file marinedrugs-22-00572-s001.zip › marinedrugs-3356271-supplementary.pdf]

Supplementary materials

Identification and Characterization of Two Aryl Sulfotransferases from Deep-Sea Marine Fungi and their Implications in the Sulfation of Secondary Metabolites

Nicolas Graziano[1], Beatriz Arce-López[1], Tristan Barbeyron[2], Ludovic Delage[2], Elise Gerometta[3], Catherine Roullier[3], Gaëtan Burgaud[1], Elisabeth Poirier[1], Laure Martinelli[4], Jean-Luc Jany[1], Nolwenn Hymery[1] and Laurence Meslet-Cladiere1*

[1] Univ Brest, INRAE, Laboratoire Universitaire de Biodiversité et Écologie Microbienne, F-29280 Plouzané, France

[2] Sorbonne Université, CNRS, Laboratory of Integrative Biology of Marine Models (LBI2M), Station Biologique de Roscoff (SBR), 29688 Roscoff cedex, Bretagne, France.

[3] Institut des Substances et Organismes de la Mer, Nantes Université, ISOMER, UR 2160, F-44000 Nantes, Bretagne, France.

[4] Department of Biochemistry, Max Planck Institute for Chemical Ecology, Hans-Knöll strasse 8, Jena 07455, Germany

* Correspondence: laurence.meslet@univ-brest.fr

Table S1: Accession number, organismal origin and Source DataBank of proteins used to build the Phylogenetic tree of putative fungal SULTs.

**Accession number**	***Species***	**Data bank**
Aspparac1|13997	*Aspergillus paraconicus DTO 356-C9*	JGI MycoCosm
Asppeni1|206091	*Aspergillus penicilloides* CBS 540.65	JGI MycoCosm
Asppuu1|241528	*Aspergillus creber* IBT 32277	JGI MycoCosm
Aspang1|939294	*Aspergillus angustatus* CBS 273.65	JGI MycoCosm
Aspcreb1|118157	*Aspergillus puulaauensis* IBT 32284	JGI MycoCosm
Aspsy1|677403	*Aspergillus sydowii* CBS 593.65	JGI MycoCosm
Aspesubv1|20345	*Aspergillus subversicolor* IBT 32280	JGI MycoCosm
Aspare1|29029	*Aspergillus arenarioides* CBS 138200	JGI MycoCosm
Pencit1|523595	*Penicillium citreonigrum* cont 1189274	JGI MycoCosm
Aspcaesp1|96920	*Aspergillus caespitosus* CBS 103.45	JGI MycoCosm
Asppsf1|171138	*Aspergillus pseudofelis* IBT 34107	JGI MycoCosm
Asppsev1|266425	*Aspergillus pseudoviridinutans* IBT 34175	JGI MycoCosm
Aspcreb1|578829	*Aspergillus creber* IBT 32277	JGI MycoCosm
Aspfre1|170413	*Aspergillus frequens* CBS 586.65	JGI MycoCosm
Aspneof1|275928	*Aspergillus neoflavipes* CBS 260.73	JGI MycoCosm
Aspfla1|251213	*Aspergillus flavipes* CBS 22552	JGI MycoCosm
Cocba1|120016	*Coccodinium bartschii* CBS 121709	JGI MycoCosm
Triei1|329372	*Trichoderma eijii* CBS 133190	JGI MycoCosm
Tricae1|295709	*Trichoderma caesareum* CBS 124369	JGI MycoCosm
Tribre1|115453	*Trichoderma brevicompactum* IBT40841	JGI MycoCosm
Tribrev1|194863	*Trichoderma brevicompactum* SZMC 28004	JGI MycoCosm
Hirmi1|5724	*Hirsutella minnesotensis* 3608	JGI MycoCosm
Corin1|51319	*Corynascella inaequalis* CBS 284.82	JGI MycoCosm
Chame1|384042	*Chaetomium* sp. MPI-CAGE-AT-0009	JGI MycoCosm
Chafi1|450908	*Chaetomidium fimeti* CBS 168.71	JGI MycoCosm
Bombar1|562487	Lasiosphaeriaceae sp. AZ0830	JGI MycoCosm
CorRAO_2017_1|129	*Cordyceps* sp. RAO 2017	JGI MycoCosm
Cylol1|461710	*Cylindrocarpon olidum* MPI-CAGE-CH-0241	JGI MycoCosm
Lashir1|656251	*Lasiosphaeris hirsuta* SMH4607-1	JGI MycoCosm
Hypros1|9646	*Hypomyces rosellus* CCMJ2808	JGI MycoCosm
Xanpa2|312449	Xanthoria parietina 46-1-SA22	JGI MycoCosm
Asptep1_1|38926	*Aspergillus teporis* DTO 058-E5	JGI MycoCosm
Aspcost1|191709	*Aspergillus costiformis* CBS 101749	JGI MycoCosm
Aspcri1|1346	*Aspergillus cristatus* GZAAS20.1005	JGI MycoCosm
Aspare1|23349	*Aspergillus arenarioides* CBS 138200	JGI MycoCosm

Aspcost1|219821	*Aspergillus costiformis* CBS 101749	JGI MycoCosm
Aspcri1|1397	*Aspergillus cristatus* GZAAS20.1005	JGI MycoCosm
Aspneos1|39605	*Aspergillus neostromatioides* CBS 265.73	JGI MycoCosm
Asppset1|282974	*Aspergillus pseudotamarii* CBS 117625	JGI MycoCosm
Aspegy1|549980	*Aspergillus egyptiacus* CBS 656.73	JGI MycoCosm
Asppul1|301049	*Aspergillus pulvericola* CBS 137327	JGI MycoCosm
Aspwest1|280934	*Aspergillus westerdijkiae* CBS 112803	JGI MycoCosm
Aspste1|424389	*Aspergillus steynii* IBT 23096	JGI MycoCosm
Aspele1|373803	*Aspergillus elegans* CBS 116.39	JGI MycoCosm
Aspave1|128849	*Aspergillus avenaceus* IBT 18842	JGI MycoCosm
Aspisr1|12166	*Aspergillus israelensis* CBS 140627	JGI MycoCosm
Aspnom13137_1	*Aspergillus nomius* NRRL 13137	JGI MycoCosm
Aspnom1|145397	*Aspergillus nomius* IBT 12657	JGI MycoCosm
Asppsen1|285275	*Aspergillus pseudonomius* CBS 119388	JGI MycoCosm
Aspneos1|28087	*Aspergillus neostromatioides* CBS 265.73	JGI MycoCosm
Aspdro1|237566	*Aspergillus dromiae* CBS 140633	JGI MycoCosm
Aspfili1|201975	*Aspergillus filifera* CBS 114510	JGI MycoCosm
Penox1|82	*Penicillium oxalicum* 114-2	JGI MycoCosm
Aspcap1|105489	*Aspergillus capensis* CBS 138188	JGI MycoCosm
Aspiiz1|334983	*Aspergillus iizukae* CBS 541.69	JGI MycoCosm
Asptem1|380815	*Aspergillus templicola* CBS 138181	JGI MycoCosm
Aspfre1|154677	*Aspergillus frequens* CBS 586.65	JGI MycoCosm
Aspneof1|281613	*Aspergillus neoflavipes* CBS 260.73	JGI MycoCosm
Aspave1|170019	*Aspergillus avenaceus* IBT 18842	JGI MycoCosm
Aspuda1|8886	*Aspergillus udagawae* IFM 46973	JGI MycoCosm
Aspudag1|340486	*Aspergillus udagawae* CBS 114217	JGI MycoCosm
Aspvir1|242959	*Aspergillus viridinutans* CBS 127.56	JGI MycoCosm
Aspsia1|500903	*Aspergillus siamensis* CBS 137452	JGI MycoCosm
Asppsf1|170059	*Aspergillus pseudofelis* IBT 34107	JGI MycoCosm
Asppara1|460256	*Aspergillus parafelis* IBT 34187	JGI MycoCosm
Pfla17421_1|361382	*Penicillium flavigenum* ITEM 17421	JGI MycoCosm
P50225	*Homo sapiens*	UniProt
P50226	*Homo sapiens*	UniProt
P0DMM9	*Homo sapiens*	UniProt
O43704	*Homo sapiens*	UniProt
O00338	*Homo sapiens*	UniProt
O75897	*Homo sapiens*	UniProt
Q6IMI6	*Homo sapiens*	UniProt

P49888	*Homo sapiens*	UniProt
Q06520	*Homo sapiens*	UniProt
O00204	*Homo sapiens*	UniProt
Q9BR01	*Homo sapiens*	UniProt
Q6IMI4	*Homo sapiens*	UniProt
Q9FG94	*Arabidiopsis thaliana*	UniProt
Q9M1V2	*Arabidiopsis thaliana*	UniProt
Q9M1V1	*Arabidiopsis thaliana*	UniProt
Q9FX56	*Arabidiopsis thaliana*	UniProt
Q9FX55	*Arabidiopsis thaliana*	UniProt
O82330	*Arabidiopsis thaliana*	UniProt
Q9STQ6	*Arabidiopsis thaliana*	UniProt
Q9FZ91	*Arabidiopsis thaliana*	UniProt
Q8RV79	*Arabidiopsis thaliana*	UniProt
P52839	*Arabidiopsis thaliana*	UniProt
Q9ZPQ5	*Arabidiopsis thaliana*	UniProt
Q8GZ53	*Arabidiopsis thaliana*	UniProt
Q8L5A7	*Arabidiopsis thaliana*	UniProt
Q9C9D0	*Arabidiopsis thaliana*	UniProt
Q9FZ80	*Arabidiopsis thaliana*	UniProt
Q9C9C9	*Arabidiopsis thaliana*	UniProt
Acastr1|124098	*Acarospora strigata* CBS 132363	JGI MycoCosm
Parmar1|550806	*Parapyrenis maritima* CBS 538.93	JGI MycoCosm
Mictr1|366783	*Microascus trigonosporus* CBS 218.31	JGI MycoCosm
AcreTS7_1|682583	*Emericellopsis* sp. TS7	JGI MycoCosm
Stagr1|521001	*Stanjemonium grisellum* CBS 655.79	JGI MycoCosm
Fusla1|8029	*Fusarium langsethiae* Fl201059	JGI MycoCosm
Fusspo1|838253	*Fusarium sporotrichioides* NRRL 3299	JGI MycoCosm
Fuspoa1|582615	*Fusarium poae* NRRL 26941	JGI MycoCosm
Fuspo1|4763	*Fusarium poae* 2516	JGI MycoCosm
Fusps1|5063	*Fusarium pseudograminearum* CS3096	JGI MycoCosm
Fuspse1|458980	*Fusarium pseudograminearum* NRRL 62612	JGI MycoCosm
Fuscu1|4912	*Fusarium culmorum* UK99	JGI MycoCosm
Fusgra3|1223073	*Fusarium graminearum* Z3639	JGI MycoCosm
Fusgr1|5332	*Fusarium graminearum* PH-1 (NRRL 31084)	JGI MycoCosm
Fusgr2_1|1411487	*Fusarium graminearum* PH-1	JGI MycoCosm
Fusven1|545136	*Fusarium venenatum* MPI-CAGE-CH-0201	JGI MycoCosm
Fussul1|372150	*Fusarium sulawesiensis* NRRL 66472	JGI MycoCosm

Fuseq1|340781	*Fusarium* sp. FIESC 12 MPI-CAGE-AA-0113	JGI MycoCosm
Fustri1|505446	*Fusarium avenaceum* MPI-SDFR-AT-0044	JGI MycoCosm
Fusave1|592239	*Fusarium avenaceum* NRRL 54939	JGI MycoCosm
Fustr1|405601	*Fusarium tricinctum* MPI-SDFR-AT-0068	JGI MycoCosm
Fusacu1|417691	*Fusarium acuminatum* F829	JGI MycoCosm
Fuslat1|189704	*Fusarium lateritium* NRRL 13622	JGI MycoCosm
Fusnis1|370335	*Fusarium nisikadoi* NRRL 25179	JGI MycoCosm
Fusma1|15630	*Fusarium nisikadoi* NRRL 25179	JGI MycoCosm
Fox26365_1|237193	*Fusarium oxysporum* NRRL 26365	JGI MycoCosm
FoxII5|11254	*Fusarium oxysporum* f.sp. *cubense* II5	JGI MycoCosm
Fusoxcub1|11254	*Fusarium oxysporum* f. sp. *cubense* tropical race 4 54006 (II5)	JGI MycoCosm
Fusfoe1|682869	*Fusarium foetens* NRRL 38302	JGI MycoCosm
Fusox32931|19015	*Fusarium oxysporum* NRRL 32931	JGI MycoCosm
FoxFo5176|303043	*Fusarium oxysporum* Fo5176	JGI MycoCosm
FopisF79_1|630488	*Fusarium oxysporum* f. sp. pisi F79	JGI MycoCosm
FopisF109_1|782868	*Fusarium oxysporum* f. sp. pisi F109	JGI MycoCosm
FopisF105_1|386494	*Fusarium oxysporum* f. sp. pisi F105	JGI MycoCosm
FopisF23_1|357295	*Fusarium oxysporum* f. sp. pisi F23	JGI MycoCosm
Fusoxcon1|25652	*Fusarium oxysporum* f. sp. conglutinans race 2 54008 (PHW808)	JGI MycoCosm
FoxPHW726_1|467400	*Fusarium oxysporum* f. sp. *matthiolae* strain PHW726	JGI MycoCosm
Fusoxalb1|612919	*Fusarium oxysporum* f. sp. *albedinis*	JGI MycoCosm
Fusoxy1|600896	*Fusarium oxysporum* MPI-SDFR-AT-0094	JGI MycoCosm
Fusoxlyc1|6420	*Fusarium oxysporum* f. sp. *lycopersici* MN25 (FoMN25) NRRL 54003	JGI MycoCosm
Fusoxrad1|20887	*Fusarium oxysporum* f. sp. *radicis-lycopersici* 26381 (CL57)	JGI MycoCosm
Fusoxmel1|9010	*Fusarium oxysporum* f. sp. *melonis* (FoMelon) NRRL 26406	JGI MycoCosm
Fusox2|16187	*Fusarium oxysporum* f. sp. lycopersici strain 4287	JGI MycoCosm
FoxF1010_1|341579	*Fusarium oxysporum* F10-10	JGI MycoCosm
FoxF1003_1|614138	*Fusarium oxysporum* F10-03	JGI MycoCosm
FoxF202_1|599566	*Fusarium oxysporum* F2-02	JGI MycoCosm
FoxF204_1|504128	*Fusarium oxysporum* F2-04	JGI MycoCosm
FoxF1008_1|863917	*Fusarium oxysporum* F10-08	JGI MycoCosm
FoxF201_1|756896	*Fusarium oxysporum* F2-01	JGI MycoCosm
FoxF1004_1|824502	*Fusarium oxysporum* F10-04	JGI MycoCosm
FoxF207_1|703083	*Fusarium oxysporum* F2-07	JGI MycoCosm

FoxF206_1|623806	*Fusarium oxysporum* F2-06	JGI MycoCosm
FusoxT415|575043	*Fusarium oxysporum* f. sp. pisi T415	JGI MycoCosm
Foxbas1|166260	*Fusarium oxysporum* f.sp. basilici Amherst-33	JGI MycoCosm
Fobas23_1|507479	*Fusarium oxysporum* f.sp. basilici Amherst-23	JGI MycoCosm
Fobas72_1|297712	*Fusarium oxysporum* f.sp. basilici Amherst-72	JGI MycoCosm
Fusoxrap1|11279	*Fusarium oxysporum* f. sp. *raphani* 54005	JGI MycoCosm
Fusoxpis1|6153	*Fusarium oxysporum* f. sp. *pisi* HDV247	JGI MycoCosm
Fusoxys1|671828	*Fusarium oxysporum* MPI-CAGE-CH-0212	JGI MycoCosm
Fusoxvas1|5432	*Fusarium oxysporum* f. sp. *vasinfectum* 25433	JGI MycoCosm
FusoxFo47_2|251013	*Fusarium oxysporum* Fo47	JGI MycoCosm
FusoxFo47|17770	*Fusarium oxysporum* Fo47	JGI MycoCosm
FoxMRL8996|447775	*Fusarium oxysporum* MRL8996	JGI MycoCosm
Fuspro1|5503	*Fusarium proliferatum* ET1	JGI MycoCosm
Fusprol1|214381	*Fusarium proliferatum* ET1	JGI MycoCosm
Fusve2|11613	*Fusarium verticillioides* 7600	JGI MycoCosm
Fusnyg1|498807	*Fusarium nygamai* NRRL 66327	JGI MycoCosm
Fusfu1|8157	*Fusarium fujikuroi* IMI 58289	JGI MycoCosm
Fuspr1|10859	*Fusarium proliferatum* NRRL 62905	JGI MycoCosm
Fusco1|502355	*Fusarium commune* MPI-SDFR-AT-0072	JGI MycoCosm
Fusre1|525610	*Fusarium redolens* MPI-CAGE-AT-0023	JGI MycoCosm
Fussub1|437068	*Fusarium subglutinans* NRRL 66333	JGI MycoCosm
Fuscon1|462882	*Fusarium concolor* NRRL 13459	JGI MycoCosm
XylFL0043|326047	*Xylaria* sp. FL0043	JGI MycoCosm
XylFL0933|360082	*Xylaria* sp. FL0933	JGI MycoCosm
XyFL0064_1|187653	*Xylaria* sp. FL0064	JGI MycoCosm
XylFL1042|334761	*Xylaria* sp. FL1042	JGI MycoCosm
Xylcas124033_1|293297	*Xylaria* cf. *castorea* CBS 124033	JGI MycoCosm
Xyltel121673_1|560746	*Xylaria telfairii* CBS 121673	JGI MycoCosm
Xylcub1|476811	*Xylaria flabelliformis* NC1011	JGI MycoCosm
Xylcube1|254516	*Xylaria cubensis* CBS 116.85	JGI MycoCosm
Hypvoc1|399191	*Xylaria flabelliformis* CMRP688	JGI MycoCosm
Xylscr1|451695	*Xylaria scruposa* CBS 123580	JGI MycoCosm
Xyllon1|583925	*Xylaria longipes* CBS 148.73	JGI MycoCosm
Xylven1|368622	*Xylaria venustula* FL0490	JGI MycoCosm
Xylarb124340_1	*Xylaria arbuscula* CBS 124340	JGI MycoCosm
XylarbFL1030|299218	*Xylaria arbuscula* FL1030	JGI MycoCosm
Xylhel2|5999	*Xylaria* cf. *heliscus* FL0509	JGI MycoCosm
Xylbam139988_1	*Xylaria bambusicola* CBS 139988	JGI MycoCosm

Astsub1|411138	*Astrocystis sublimbata* CBS 130006	JGI MycoCosm
Xyldig1|419681	*Xylaria digitata* CBS 161.22	JGI MycoCosm
Fusdim1|366924	*Fusarium dimerum* NRRL 20691	JGI MycoCosm
Micbo1|209143	*Microdochium bolleyi* J235TASD1	JGI MycoCosm
Micbo1|218045	*Microdochium bolleyi* J235TASD1	JGI MycoCosm
Mictri1|239161	*Microdochium trichocladiopsis* MPI-CAGE-CH-0230	JGI MycoCosm
Mictri1|372541	*Microdochium trichocladiopsis* MPI-CAGE-CH-0230	JGI MycoCosm
Mictri1|274693	*Microdochium trichocladiopsis* MPI-CAGE-CH-0230	JGI MycoCosm
Acrst1|1366428	*Acremonium strictum* DS1bioAY4a	JGI MycoCosm
Sarstr1|328400	*Sarocladium strictum* UNK	JGI MycoCosm
Acrst1|1397794	*Acremonium strictum* DS1bioAY4a	JGI MycoCosm
Sarstr1|228448	*Sarocladium strictum* UNK	JGI MycoCosm
XylFL0255|383015	*Xylariaceae* sp. FL0255	JGI MycoCosm
XylFL1019|271057	*Xylariaceae* sp. FL1019	JGI MycoCosm
XyFL1272_2|506836	Xylariaceae sp. FL1272	JGI MycoCosm
XyFL1272_2|107782	Xylariaceae sp. FL1272	JGI MycoCosm
Aurpu_var_mel1|61258	*Aureobasidium pullulans* var. *melanogenum* CBS 110374	JGI MycoCosm
Horwer1|684	*Hortaea werneckii* EXF-2000	JGI MycoCosm
Horwer1|1409	*Hortaea werneckii* EXF-2000 M0	JGI MycoCosm

Table S2: Accession number, organismal origin and Source DataBank of proteins used to build the Phylogenetic tree of St1 SULTs family (PF00685).

Accesion number	*Species*	Data bank
Q7ZUS4	*Danio rerio*	UniProt
Q6DHG7	*Danio rerio*	UniProt
Q7T2V2	*Danio rerio*	UniProt
Q6PH37	*Danio rerio*	UniProt
A0A0A6YYL2	*Homo sapiens*	UniProt
Q1ET64	*Homo sapiens*	UniProt
Q1ET61	*Homo sapiens*	UniProt
P0DMN0	*Homo sapiens*	UniProt
Q2TAB3	*Homo sapiens*	UniProt
Q1ET62	*Homo sapiens*	UniProt
P50225	*Homo sapiens*	UniProt
A0A024QZB4	*Homo sapiens*	UniProt
P50226	*Homo sapiens*	UniProt
P52846	*Macaca fascicularis*	UniProt
Q29476	*Canis lupus*	UniProt
P50227	*Bos taurus*	UniProt
P52840	*Mus musculus*	UniProt
A0A151P233	*Alligator mississippiensis*	UniProt
P49891	*Mus musculus*	UniProt
Q53X91	*Homo sapiens*	UniProt
P19217	*Bos taurus*	UniProt
P49887	*Cavia porcellus*	UniProt
A0A151MJ36	*Alligator mississippiensis*	UniProt
F1NMA2	*Gallus gallus*	UniProt
Q3UZZ6	*Mus musculus*	UniProt
A0A151MIY0	*Alligator mississippiensis*	UniProt
A0A151MIZ5	*Alligator mississippiensis*	UniProt
A0A151MIX3	*Alligator mississippiensis*	UniProt
Q8JG30	*Gallus gallus*	UniProt
P52847	*Rattus norvegicus*	UniProt
Q95JD5	*Canis lupus*	UniProt
Q3T0Y3	*Bos taurus*	UniProt
Q7SZ93	*Xenopus laevis*	UniProt
Q6DJL6	*Xenopus laevis*	UniProt

Q4FZP1	*Xenopus laevis*	UniProt
Q6GP49	*Xenopus laevis*	UniProt
Q80VR3	*Mus musculus*	UniProt
Q6IMI6	*Homo sapiens*	UniProt
Q90WR6	*Gallus gallus*	UniProt
A0A151NWU4	*Alligator mississippiensis*	UniProt
O75897	*Homo sapiens*	UniProt
A0A2K5X3B6	*Macaca fascicularis*	UniProt
B7ZSE0	*Xenopus laevis*	UniProt
A8E5Z0	*Xenopus laevis*	UniProt
Q5U5E7	*Xenopus laevis*	UniProt
Q66KW4	*Xenopus laevis*	UniProt
Q9WUW8	*Rattus norvegicus*	UniProt
Q9D939	*Mus musculus*	UniProt
O00338	*Homo sapiens*	UniProt
B8ZZF7	*Homo sapiens*	UniProt
O46503	*Oryctolagus cuniculus*	UniProt
Q5EAW0	*Xenopus laevis*	UniProt
A0A8V0XP18	*Gallus gallus*	UniProt
A0A151MJB9	*Alligator mississippiensis*	UniProt
A0A151MYA6	*Alligator mississippiensis*	UniProt
A5D8P4	*Xenopus laevis*	UniProt
B3KT14	*Homo sapiens*	UniProt
B4DLP0	*Homo sapiens*	UniProt
B7ZSE1	*Xenopus laevis*	UniProt
F1RD72	*Danio rerio*	UniProt
G3V9R3	*Rattus norvegicus*	UniProt
H3BRY5	*Homo sapiens*	UniProt
O43704	*Homo sapiens*	UniProt
P0DMM9	*Homo sapiens*	UniProt
P17988	*Rattus norvegicus*	UniProt
P49888	*Homo sapiens*	UniProt
P50237	*Rattus norvegicus*	UniProt
P52844	*Rattus norvegicus*	UniProt
Q1ET63	*Homo sapiens*	UniProt
Q1ET65	*Homo sapiens*	UniProt
Q49IK6	*Danio rerio*	UniProt

Q68EV4	*Xenopus laevis*	UniProt
Q6DHH8	*Danio rerio*	UniProt
Q6XZC1	*Danio rerio*	UniProt
Q7ZYH0	*Xenopus laevis*	UniProt
Q9QWG7	*Mus musculus*	UniProt
Q9WUW9	*Rattus norvegicus*	UniProt
A0A974C2H4	*Xenopus laevis*	UniProt
A0A974C5W8	*Xenopus laevis*	UniProt
A0A8M3AVI4	*Danio rerio*	UniProt
A0A8M9PQS2	*Danio rerio*	UniProt
Q0IHC7	*Xenopus laevis*	UniProt
A0A1L8GEQ4	*Xenopus laevis*	UniProt
F1QV04	*Danio rerio*	UniProt
Q08C30	*Danio rerio*	UniProt
A0A8V0XZY5	*Gallus gallus*	UniProt
Q1L872	*Danio rerio*	UniProt
Q1L875	*Danio rerio*	UniProt
Q1L874	*Danio rerio*	UniProt
F1QPA8	*Danio rerio*	UniProt
A0A1L8G8Y7	*Xenopus laevis*	UniProt
A0A1L8G9B9	*Xenopus laevis*	UniProt
O46640	*Oryctolagus cuniculus*	UniProt
A0A151M2Z2	*Alligator mississippiensis*	UniProt
A0A151M2Y6	*Alligator mississippiensis*	UniProt
A0A1L8G2T9	*Xenopus laevis*	UniProt
Q1L873	*Danio rerio*	UniProt
Q6IR96	*Xenopus laevis*	UniProt
O35403	*Mus musculus*	UniProt
A0A151M2Y1	*Alligator mississippiensis*	UniProt
A0A3Q2U398	*Gallus gallus*	UniProt
P50236	*Mus musculus*	UniProt
B2RVI8	*Mus musculus*	UniProt
P52843	*Mus musculus*	UniProt
P22789	*Rattus norvegicus*	UniProt
P50235	*Rattus norvegicus*	UniProt
P52841	*Cavia porcellus*	UniProt
Q06520	*Homo sapiens*	UniProt

P52842	*Macaca fascicularis*	UniProt
Q8BGL3	*Mus musculus*	UniProt
O35400	*Mus musculus*	UniProt
O00204	*Homo sapiens*	UniProt
Q66KH3	*Xenopus laevis*	UniProt
A0A151NKZ4	*Alligator mississippiensis*	UniProt
A0A151NKZ8	*Alligator mississippiensis*	UniProt
A0A151NL14	*Alligator mississippiensis*	UniProt
A0A151MG72	*Alligator mississippiensis*	UniProt
A0A1L8FNN9	*Xenopus laevis*	UniProt
A0A1L8FNR0	*Xenopus laevis*	UniProt
Q6INL4	*Xenopus laevis*	UniProt
A1L2N2	*Xenopus laevis*	UniProt
P15709	*Rattus norvegicus*	UniProt
P50234	*Cavia porcellus*	UniProt
A8K015	*Homo sapiens*	UniProt
Q29YR5	*Rattus norvegicus*	UniProt
Q5M7C2	*Xenopus laevis*	UniProt
A0A151P523	*Alligator mississippiensis*	UniProt
A1L3M6	*Xenopus laevis*	UniProt
A0A1L8FGT8	*Xenopus laevis*	UniProt
A0A974HAK9	*Xenopus laevis*	UniProt
A0A8J1LDD2	*Xenopus laevis*	UniProt
A0A974CH11	*Xenopus laevis*	UniProt
A0A8J1LEX3	*Xenopus laevis*	UniProt
A0A8V0XP41	*Gallus gallus*	UniProt
A0A8V0XIP1	*Gallus gallus*	UniProt
A0A8V0XIS1	*Gallus gallus*	UniProt
A0A8V0XLB2	*Gallus gallus*	UniProt
A0A8V0XEJ6	*Gallus gallus*	UniProt
Q6P2U4	*Danio rerio*	UniProt
E7F734	*Danio rerio*	UniProt
A0EXC5	*Danio rerio*	UniProt
A4FUP0	*Danio rerio*	UniProt
A9UND3	*Drosophila melanogaster*	UniProt
Q9W1L7	*Drosophila melanogaster*	UniProt
Q8MRH3	*Drosophila melanogaster*	UniProt

A0A0B4KF40	*Drosophila melanogaster*	UniProt
Q9W1L8	*Drosophila melanogaster*	UniProt
Q6NL86	*Drosophila melanogaster*	UniProt
B7YZP4	*Drosophila melanogaster*	UniProt
Q9BR01	*Homo sapiens*	UniProt
A0A024R4X9	*Homo sapiens*	UniProt
P63047	*Rattus norvegicus*	UniProt
E1C8B2	*Gallus gallus*	UniProt
Q52KW3	*Xenopus laevis*	UniProt
Q68EV3	*Xenopus laevis*	UniProt
Q1RLU6	*Danio rerio*	UniProt
B7Z320	*Homo sapiens*	UniProt
P63046	*Mus musculus*	UniProt
A0A151N6I9	*Alligator mississippiensis*	UniProt
A0A974D6E1	*Xenopus laevis*	UniProt
A0A974DA59	*Xenopus laevis*	UniProt
A0A1L8GQ73	*Xenopus laevis*	UniProt
A0A151NIY3	*Alligator mississippiensis*	UniProt
B7ZWN4	*Mus musculus*	UniProt
A1L2M9	*Xenopus laevis*	UniProt
A0A8V0YIL1	*Gallus gallus*	UniProt
K1QDM6	*Crassostrea gigas*	UniProt
K1QY34	*Crassostrea gigas*	UniProt
K1P7I4	*Crassostrea gigas*	UniProt
K1PV91	*Crassostrea gigas*	UniProt
K1PCB5	*Crassostrea gigas*	UniProt
K1PF38	*Crassostrea gigas*	UniProt
K1P8Y3	*Crassostrea gigas*	UniProt
K1PIV5	*Crassostrea gigas*	UniProt
K1QTG7	*Crassostrea gigas*	UniProt
K1RFK5	*Crassostrea gigas*	UniProt
K1PPS3	*Crassostrea gigas*	UniProt
K1RHM0	*Crassostrea gigas*	UniProt
K1PZI9	*Crassostrea gigas*	UniProt
K1QLP7	*Crassostrea gigas*	UniProt
K1RV76	*Crassostrea gigas*	UniProt
K1PR19	*Crassostrea gigas*	UniProt

K1PMV7	*Crassostrea gigas*	UniProt
K1PZN7	*Crassostrea gigas*	UniProt
K1PHZ9	*Crassostrea gigas*	UniProt
K1Q6V1	*Crassostrea gigas*	UniProt
Q6GM55	*Xenopus laevis*	UniProt
Q3KPL6	*Xenopus laevis*	UniProt
A0A1L8G220	*Xenopus laevis*	UniProt
A0A1L8G7X4	*Xenopus laevis*	UniProt
Q0IH61	*Xenopus laevis*	UniProt
Q5BJ35	*Xenopus laevis*	UniProt
A0A1L8G274	*Xenopus laevis*	UniProt
A0A151MHD3	*Alligator mississippiensis*	UniProt
Q6IMI4	*Homo sapiens*	UniProt
Q6WG18	*Pan troglodytes*	UniProt
P0CC03	*Mus musculus*	UniProt
A0A1L1RLU1	*Gallus gallus*	UniProt
A0A151M2J5	*Alligator mississippiensis*	UniProt
A0A151M2N4	*Alligator mississippiensis*	UniProt
F1QYJ6	*Danio rerio*	UniProt
Q569S3	*Xenopus laevis*	UniProt
Q6GN52	*Xenopus laevis*	UniProt
A0A0C4DG03	*Homo sapiens*	UniProt
Q6WG17	*Pan troglodytes*	UniProt
A0A151MH80	*Alligator mississippiensis*	UniProt
A0A151M2H9	*Alligator mississippiensis*	UniProt
A0A151M2I3	*Alligator mississippiensis*	UniProt
O57338	*Gallus gallus*	UniProt
A0A974HJA9	*Xenopus laevis*	UniProt
A0A974CW61	*Xenopus laevis*	UniProt
A0A8J1KQT4	*Xenopus laevis*	UniProt
A0A974HH65	*Xenopus laevis*	UniProt
A0A8V0XZJ5	*Gallus gallus*	UniProt
A0A8V0XVW0	*Gallus gallus*	UniProt
A0A8V0XVW4	*Gallus gallus*	UniProt
A0A8V0XLT1	*Gallus gallus*	UniProt
A0A8V0XT36	*Gallus gallus*	UniProt
A0A974CUZ1	*Xenopus laevis*	UniProt

A0A974HHD8	*Xenopus laevis*	UniProt
F6QP54	*Ciona intestinalis*	UniProt
F6ZYZ9	*Ciona intestinalis*	UniProt
F6ZAA5	*Ciona intestinalis*	UniProt
F6ZNS9	*Ciona intestinalis*	UniProt
H2Y2F5	*Ciona intestinalis*	UniProt
F6XHN7	*Ciona intestinalis*	UniProt
F6TLK5	*Ciona intestinalis*	UniProt
F6STM9	*Ciona intestinalis*	UniProt
H2XPA6	*Ciona intestinalis*	UniProt
F6Y7J1	*Ciona intestinalis*	UniProt
F6SQW0	*Ciona intestinalis*	UniProt
F6ZNT5	*Ciona intestinalis*	UniProt
F6XHS1	*Ciona intestinalis*	UniProt
A0A7M7P2G7	*Strongylocentrotus purpuratus*	UniProt
A0A7M7N0B5	*Strongylocentrotus purpuratus*	UniProt
A0A7M7P085	*Strongylocentrotus purpuratus*	UniProt
A0A7M7PQ65	*Strongylocentrotus purpuratus*	UniProt
A0A7M7HPT0	*Strongylocentrotus purpuratus*	UniProt
A0A7M7MXA6	*Strongylocentrotus purpuratus*	UniProt
A0A7M7PI38	*Strongylocentrotus purpuratus*	UniProt
A0A7M7PK18	*Strongylocentrotus purpuratus*	UniProt
A0A7M7P5H0	*Strongylocentrotus purpuratus*	UniProt
A0A7M7REA6	*Strongylocentrotus purpuratus*	UniProt
A0A7M7P013	*Strongylocentrotus purpuratus*	UniProt
A0A7M7RFM2	*Strongylocentrotus purpuratus*	UniProt
A0A7M7PLD2	*Strongylocentrotus purpuratus*	UniProt
A0A7M7PMB5	*Strongylocentrotus purpuratus*	UniProt
A0A7M7PK02	*Strongylocentrotus purpuratus*	UniProt
A0A7M7RD60	*Strongylocentrotus purpuratus*	UniProt
A0A7M7HIX3	*Strongylocentrotus purpuratus*	UniProt
A0A7M7P704	*Strongylocentrotus purpuratus*	UniProt
A0A7M7HF07	*Strongylocentrotus purpuratus*	UniProt
A0A7M7RFD3	*Strongylocentrotus purpuratus*	UniProt
A0A7M7NIF6	*Strongylocentrotus purpuratus*	UniProt
A0A7M7NI76	*Strongylocentrotus purpuratus*	UniProt
A0A7M7NI67	*Strongylocentrotus purpuratus*	UniProt

A0A3M6U731	*Pocillopora damicornis*	UniProt
A0A3M6U6I1	*Pocillopora damicornis*	UniProt
A7S682	*Nematostella vectensis*	UniProt
A0A3M6UFC9	*Pocillopora damicornis*	UniProt
A0A3M6UG75	*Pocillopora damicornis*	UniProt
A0A3M6U6U7	*Pocillopora damicornis*	UniProt
A0A3M6V496	*Pocillopora damicornis*	UniProt
A0A3M6V496	*Pocillopora damicornis*	UniProt
A0A1X7V4E9	*Amphimedon queenslandica*	UniProt
A0A1X7V2D8	*Amphimedon queenslandica*	UniProt
A0A1X7V4E2	*Amphimedon queenslandica*	UniProt
A0A1X7TID1	*Amphimedon queenslandica*	UniProt
A0A1X7VMC9	*Amphimedon queenslandica*	UniProt
A0A1X7SYG2	*Amphimedon queenslandica*	UniProt
A0A1X7UT16	*Amphimedon queenslandica*	UniProt
A0A1X7T295	*Amphimedon queenslandica*	UniProt
A0A1X7VU44	*Amphimedon queenslandica*	UniProt
A0A1X7VFD4	*Amphimedon queenslandica*	UniProt
A0A1X7UQM3	*Amphimedon queenslandica*	UniProt
A0A1X7US23	*Amphimedon queenslandica*	UniProt
A0A1X7V7Q0	*Amphimedon queenslandica*	UniProt
A0A1X7UF11	*Amphimedon queenslandica*	UniProt
A0A1X7UJA4	*Amphimedon queenslandica*	UniProt
A0A1X7US86	*Amphimedon queenslandica*	UniProt
A0A1X7USM6	*Amphimedon queenslandica*	UniProt
A0A1X7URA8	*Amphimedon queenslandica*	UniProt
A0A1X7UYV1	*Amphimedon queenslandica*	UniProt
A0A1X7VVH0	*Amphimedon queenslandica*	UniProt
A0A1X7VH04	*Amphimedon queenslandica*	UniProt
L7N0U7	*Ciona intestinalis*	UniProt
F6X245	*Ciona intestinalis*	UniProt
B8C826	*Thalassiosira pseudonana*	UniProt
F7BDB7	*Ciona intestinalis*	UniProt
F6X329	*Ciona intestinalis*	UniProt
B8BYU0	*Thalassiosira pseudonana*	UniProt
B7S463	*Phaeodactylum tricornutum*	UniProt
B7GCE2	*Phaeodactylum tricornutum*	UniProt

B7GCE3	*Phaeodactylum tricornutum*	UniProt
B8BUD5	*Thalassiosira pseudonana*	UniProt
B7G250	*Phaeodactylum tricornutum*	UniProt
B8CES4	*Thalassiosira pseudonana*	UniProt
B5Y457	*Phaeodactylum tricornutum*	UniProt
B7FUK0	*Phaeodactylum tricornutum*	UniProt
D7G0M1	*Ectocarpus* sp. 7	UniProt
D8LJX5	*Ectocarpus* sp. 7	UniProt
D7G187	*Ectocarpus* sp. 7	UniProt
D8LHM0	*Ectocarpus* sp. 7	UniProt
GENE_019207	*Saccharina japonica*	Phaeoexplorer database
GENE_026961	*Saccharina japonica*	Phaeoexplorer database
XLOC_024652	*Saccharina japonica*	Phaeoexplorer database
XLOC_016866	*Saccharina japonica*	Phaeoexplorer database
GENE_016729	*Saccharina japonica*	Phaeoexplorer database
GENE_009569	*Saccharina japonica*	Phaeoexplorer database
SJ10094	*Saccharina japonica*	Phaeoexplorer database
SJ19194	*Saccharina japonica*	Phaeoexplorer database
NDg5771	*Nemacystus decipiens*	Phaeoexplorer database
Cok_S_s031_6480	*Cladosiphon okamuranus*	Phaeoexplorer database
SJ05841	*Saccharina japonica*	Phaeoexplorer database
NDg3713	*Nemacystus decipiens*	Phaeoexplorer database
NDg12273.t1	*Nemacystus decipiens*	Phaeoexplorer database
NDg5820.t1	*Nemacystus decipiens*	Phaeoexplorer database
SJ06276	*Saccharina japonica*	Phaeoexplorer database
NDg3754.t1	*Nemacystus decipiens*	Phaeoexplorer database
EsiAST6	*Ectocarpus* sp. 7	Phaeoexplorer database
A0A6H5K098	*Ectocarpus* sp.	UniProt

A0A2I2GAR1	*Aspergillus steynii*	UniProt
S7Z901	*Penicillium oxalicum*	UniProt
A0A1L9T6E9	*Aspergillus sydowii*	UniProt
A0A1L9TUA4	*Aspergillus sydowii*	UniProt
Xanpa1|12063	*Xanthoria parietina*	JGI MycoCosm
A1Z9J8	*Drosophila melanogaster*	UniProt
Q7PXJ0	*Anopheles gambiae*	UniProt
R7QHS2	*Chondrus crispus*	UniProt
R7Q533	*Chondrus crispus*	UniProt
A0A5J4ZAZ9	*Porphyridium purpureum*	UniProt
B7FXZ9	*Phaeodactylum tricornutum*	UniProt
A9USC3	*Monosiga brevicollis*	UniProt
W7TT65	*Nannochloropsis gaditana*	UniProt
L1JCJ6	*Guillardia theta*	UniProt
R1DA94	*Emiliania huxleyi*	UniProt
R1DQQ4	*Emiliania huxleyi*	UniProt
R1E9C7	*Emiliania huxleyi*	UniProt
A4RZY0	*Ostreococcus lucimarinus*	UniProt
D0LN96	*Haliangium ochraceum*	UniProt
R7QN52	*Chondrus crispus*	UniProt
D0LK90	*Haliangium ochraceum*	UniProt
R1DIW8	*Emiliania huxleyi*	UniProt
R1BR92	*Emiliania huxleyi*	UniProt
UM022_0124.1	*Ulva mutabilis*	ORCAE database
L1K3Z7	*Guillardia theta*	UniProt
L1K0T0	*Guillardia theta*	UniProt
L1I9A7	*Guillardia theta*	UniProt
R1G401	*Emiliania huxleyi*	UniProt
R1D1G0	*Emiliania huxleyi*	UniProt
A0A1H1NY94	*Actinopolymorpha singaporensis*	UniProt
A1KIG2	*Mycobacterium bovis*	UniProt
A0A1X4NCW8	*Marivita geojedonensis*	UniProt
A0A0G4F3C5	*Vitrella brassicaformis*	UniProt
A0A0G4EPW9	*Vitrella brassicaformis*	UniProt
A0A0G4FKB3	*Vitrella brassicaformis*	UniProt
D0LVM1	*Haliangium ochraceum*	UniProt
A0A0C5VJ02	*Gynuella sunshinyii*	UniProt

A0A0C5W4V0	*Gynuella sunshinyii*	UniProt
A0A4R4Z872	*Nonomuraea terrae*	UniProt
P9WGB9	*Mycobacterium tuberculosis*	UniProt
A0A0G4ELR1	*Vitrella brassicaformis*	UniProt
A0A1Z3HRJ4	*Halomicronema hongdechloris*	UniProt
A0A074VEZ5	*Aureobasidium melanogenum*	UniProt
A0A074VEZ5	*Aureobasidium melanogenum*	UniProt
A0A1Z5T5L4	*Hortaea werneckii*	UniProt
S0E772	*Fusarium fujikuroi*	UniProt
A0A1Z5T779	*Hortaea werneckii*	UniProt
A0A0J9W5F8	*Fusarium oxysporum*	UniProt
Acrst1|1397794	*Sarocladium strictum*	JGI MycoCosm
Acrst1|1366428	*Sarocladium strictum*	JGI MycoCosm
A0A654FCW4	*Arabidopsis thaliana*	UniProt
A0A1I9LRV5	*Arabidopsis thaliana*	UniProt
A0A1I9LRV6	*Arabidopsis thaliana*	UniProt
A0A654FHB7	*Arabidopsis thaliana*	UniProt
A0A654EY64	*Arabidopsis thaliana*	UniProt
A0A5S9X220	*Arabidopsis thaliana*	UniProt
A0A078GZC1	*Brassica napus*	UniProt
A0A078H3L8	*Brassica napus*	UniProt
A0A078J9X5	*Brassica napus*	UniProt
A0A078GGJ8	*Brassica napus*	UniProt
Q8LEH6	*Arabidopsis thaliana*	UniProt
A0A654EK73	*Arabidopsis thaliana*	UniProt
A0A5S9WY45	*Arabidopsis thaliana*	UniProt
O82330	*Arabidopsis thaliana*	UniProt
Q9FX55	*Arabidopsis thaliana*	UniProt
A0A178WEF5	*Arabidopsis thaliana*	UniProt
A0A5S9U6R9	*Arabidopsis thaliana*	UniProt
A0A5S9XW03	*Arabidopsis thaliana*	UniProt
Q9STQ6	*Arabidopsis thaliana*	UniProt
A0A654FSX3	*Arabidopsis thaliana*	UniProt
A0A078HXZ6	*Brassica napus*	UniProt
A0A078H7T9	*Brassica napus*	UniProt
A0A654EDM3	*Arabidopsis thaliana*	UniProt
A0A5S9W5F8	*Arabidopsis thaliana*	UniProt

A0A178W8F3	*Arabidopsis thaliana*	UniProt
A0A078FEP9	*Brassica napus*	UniProt
A0A078F3D6	*Brassica napus*	UniProt
A0A078FAB3	*Brassica napus*	UniProt
Q9FG94	*Arabidopsis thaliana*	UniProt
A0A5S9YBT9	*Arabidopsis thaliana*	UniProt
A0A178UBJ0	*Arabidopsis thaliana*	UniProt
A0A078J3G1	*Brassica napus*	UniProt
A0A078IKT9	*Brassica napus*	UniProt
A0A078GW80	*Brassica napus*	UniProt
A0A178VL58	*Arabidopsis thaliana*	UniProt
A0A654EWM0	*Arabidopsis thaliana*	UniProt
A0A078G9Z0	*Brassica napus*	UniProt
A0A5S9WWW5	*Arabidopsis thaliana*	UniProt
A0A078H5J0	*Brassica napus*	UniProt
O82410	*Brassica napus*	UniProt
A0A654ERP0	*Arabidopsis thaliana*	UniProt
A0A078I8I1	*Brassica napus*	UniProt
Q6T261	*Brassica napus*	UniProt
A0A078FMH3	*Brassica napus*	UniProt
Q8L5A7	*Arabidopsis thaliana*	UniProt
A0A654FZ18	*Arabidopsis thaliana*	UniProt
Q8GZ53	*Arabidopsis thaliana*	UniProt
A0A5S9Y2X0	*Arabidopsis thaliana*	UniProt
B6TRN9	*Zea mays*	UniProt
A0A3L6F574	*Zea mays*	UniProt
A0A3L6FIN7	*Zea mays*	UniProt
A0A3L6DYT1	*Zea mays*	UniProt
A0A3L6FF19	*Zea mays*	UniProt
A0A3L6FF07	*Zea mays*	UniProt
A0A3L6FKM0	*Zea mays*	UniProt
B6TGE9	*Zea mays*	UniProt
P52835	*Flaveria bidentis*	UniProt
A0A078IDA4	*Brassica napus*	UniProt
A0A384KIF3	*Arabidopsis thaliana*	UniProt
M1FZV5	*Arabidopsis thaliana*	UniProt
A0A0K1H0V2	*Brassica napus*	UniProt

A0A078GJN3	*Brassica napus*	UniProt
A0A078FI33	*Brassica napus*	UniProt
A0A078FER1	*Brassica napus*	UniProt
A0A078HT16	*Brassica napus*	UniProt
A0A078GVH9	*Brassica napus*	UniProt
T1P4W7	*Arabidopsis thaliana*	UniProt
T1P4S7	*Arabidopsis thaliana*	UniProt
T1P4U2	*Arabidopsis thaliana*	UniProt
T1P4W6	*Arabidopsis thaliana*	UniProt
A0A078C8A1	*Brassica napus*	UniProt
B3GN18	*Arabidopsis thaliana*	UniProt
M1EU36	*Arabidopsis thaliana*	UniProt
A0A654EQH9	*Arabidopsis thaliana*	UniProt
A0A178WK39	*Arabidopsis thaliana*	UniProt
Q9C9D0	*Arabidopsis thaliana*	UniProt
A0A0K1H0Z2	*Brassica napus*	UniProt
Q9M1V1	*Arabidopsis thaliana*	UniProt
A0A178VFN5	*Arabidopsis thaliana*	UniProt
A0A5S9XI94	*Arabidopsis thaliana*	UniProt
A0A178VKH4	*Arabidopsis thaliana*	UniProt
Q9M1V2	*Arabidopsis thaliana*	UniProt
Q8RUC1	*Arabidopsis thaliana*	UniProt
A0A078I9T1	*Brassica napus*	UniProt
A0A078HB23	*Brassica napus*	UniProt
A0A078HAF1	*Brassica napus*	UniProt
Q9FX56	*Arabidopsis thaliana*	UniProt
A0A178W8T8	*Arabidopsis thaliana*	UniProt
A0A178VXW9	*Arabidopsis thaliana*	UniProt
A0A654ESY5	*Arabidopsis thaliana*	UniProt
A0A654EEI3	*Arabidopsis thaliana*	UniProt
A0A178V1D4	*Arabidopsis thaliana*	UniProt
A0A078HA80	*Brassica napus*	UniProt
Q9FZ91	*Arabidopsis thaliana*	UniProt
A0A078FH03	*Brassica napus*	UniProt
A0A078FED3	*Brassica napus*	UniProt
A0A654G7R2	*Arabidopsis thaliana*	UniProt
A0A078I6H1	*Brassica napus*	UniProt

A0A078I126	*Brassica napus*	UniProt
A0A078HX67	*Brassica napus*	UniProt
Q8RV79	*Arabidopsis thaliana*	UniProt
A0A078G6V4	*Brassica napus*	UniProt
Q9ZPQ5	*Arabidopsis thaliana*	UniProt
A0A178VSF0	*Arabidopsis thaliana*	UniProt
A0A078IYK3	*Brassica napus*	UniProt
O82408	*Brassica napus*	UniProt
A0A078IXS8	*Brassica napus*	UniProt
A0A078JHN3	*Brassica napus*	UniProt
A0A078JMQ5	*Brassica napus*	UniProt
A0A078J6I6	*Brassica napus*	UniProt
A0A078IVW1	*Brassica napus*	UniProt
P52839	*Arabidopsis thaliana*	UniProt
A0A178VZN3	*Arabidopsis thaliana*	UniProt
O82409	*Brassica napus*	UniProt
A0A078J6N1	*Brassica napus*	UniProt
A0A078JRY7	*Brassica napus*	UniProt
A0A078J0T5	*Brassica napus*	UniProt
A0A178UG65	*Arabidopsis thaliana*	UniProt
A0A178U8M1	*Arabidopsis thaliana*	UniProt
A0A317YE26	*Zea mays*	UniProt
A0A3L6D6E6	*Zea mays*	UniProt
A0A1D6Q726	*Zea mays*	UniProt
B6TEY8	*Zea mays*	UniProt
B4FVM2	*Zea mays*	UniProt
Q0DAU1	*Oryza sativa*	UniProt
Q0JNA1	*Oryza sativa*	UniProt
B6STJ6	*Zea mays*	UniProt
Q53QR3	*Oryza sativa*	UniProt
B4FQ75	*Zea mays*	UniProt
B6UIM9	*Zea mays*	UniProt
B4FSB6	*Zea mays*	UniProt
A0A1D6N1I0	*Zea mays*	UniProt
P52836	*Flaveria chlorifolia*	UniProt
P52837	*Flaveria chlorifolia*	UniProt
P52838	*Flaveria bidentis*	UniProt

A0A078IGQ2	*Brassica napus*	UniProt
A0A0K1H116	*Brassica napus*	UniProt
A0A078F9D9	*Brassica napus*	UniProt
M1FZP1	*Arabidopsis thaliana*	UniProt
Q9FZ80	*Arabidopsis thaliana*	UniProt
B3GN19	*Arabidopsis thaliana*	UniProt
A0A078G1N7	*Brassica napus*	UniProt
A0A078HT74	*Brassica napus*	UniProt
A0A078IA33	*Brassica napus*	UniProt
A0A078HPK4	*Brassica napus*	UniProt
A0A078GVI3	*Brassica napus*	UniProt
A0A078HPY5	*Brassica napus*	UniProt
T1P4V4	*Arabidopsis thaliana*	UniProt
Q9C9C9	*Arabidopsis thaliana*	UniProt
A0A654ENT6	*Arabidopsis thaliana*	UniProt
A0A178WNK0	*Arabidopsis thaliana*	UniProt
B2LU29	*Arabidopsis thaliana*	UniProt
A0A078E3L3	*Brassica napus*	UniProt
A0A078I7J9	*Brassica napus*	UniProt
A0A078JS01	*Brassica napus*	UniProt
A0A5S9WU88	*Arabidopsis thaliana*	UniProt
M1EX44	*Arabidopsis thaliana*	UniProt
A9WF11	*Chloroflexus aurantiacus*	UniProt
D7FV63	*Ectocarpus* sp. 7	UniProt
A0A1Z3HH68	*Halomicronema hongdechloris*	UniProt
B8EJV7	*Methylocella silvestris*	UniProt
A0YJZ1	*Lyngbya* sp.	UniProt
A0A1Z3HMR2	*Halomicronema hongdechloris*	UniProt
W7TKP3	*Nannochloropsis gaditana*	UniProt
B8C3T6	*Thalassiosira pseudonana*	UniProt
R1BL88	*Emiliania huxleyi*	UniProt
Q8KLM3	*Streptomyces toyocaensis*	UniProt
A0A429AFB8	*Nonomuraea* sp.	UniProt
A0A429FDT9	*Streptomyces* sp.	UniProt
A0A8H9IUR2	*Amycolatopsis bartoniae*	UniProt
A0A8H9IZE9	*Amycolatopsis bartoniae*	UniProt
B7T1D7	*uncultured soil bacterium*	UniProt

B7T1D8	*uncultured soil bacterium*	UniProt
B7T1D9	*uncultured soil bacterium*	UniProt
S4TVE7	*Streptomyces sp.*	UniProt
S5TNE2	*uncultured bacterium*	UniProt
S5UBM4	*uncultured bacterium*	UniProt
V9NHS5	*Streptomyces* sp.	UniProt
Q3HYK0	*Strongylocentrotus purpuratus*	UniProt
A0A7M7P361	*Strongylocentrotus purpuratus*	UniProt
R1CMR8	*Emiliania huxleyi*	UniProt
R1B713	*Emiliania huxleyi*	UniProt
R1DFD6	*Emiliania huxleyi*	UniProt
F4XU35	*Moorena producens*	UniProt
T2MI09	*Hydra vulgaris*	UniProt
Q9U2Z2	*Caenorhabditis elegans*	UniProt
H2Y252	*Ciona intestinalis*	UniProt
A0A7M7HF01	*Strongylocentrotus purpuratus*	UniProt
C3XSB6	*Branchiostoma floridae*	UniProt
K1S562	*Crassostrea gigas*	UniProt
Q9VHH0	*Drosophila melanogaster*	UniProt
R1CX47	*Emiliania huxleyi*	UniProt
R1C2S7	*Emiliania huxleyi*	UniProt
R1C876	*Emiliania huxleyi*	UniProt
A0A7M7RF06	*Strongylocentrotus purpuratus*	UniProt
F6W949	*Ciona intestinalis*	UniProt
R1BU78	*Emiliania huxleyi*	UniProt
R1CI51	*Emiliania huxleyi*	UniProt
X6P003	*Reticulomyxa filosa*	UniProt
F4XRC3	*Moorena producens*	UniProt
D0LKW4	*Haliangium ochraceum*	UniProt
Q86IU8	*Dictyostelium discoideum*	UniProt
F1A2V5	*Dictyostelium purpureum*	UniProt

Figure S1: Percent identity matrix of multiple sequence alignment between *Hortaea werneckii UBOCC-A*-208029 putative SULT, *Fusarium graminearum* PH-1 *Fg*SULT1, *Aspergillus sydowii* UBOCC-A- 108050 putative SULT, Human SULT1B1, SULT1C2, SULT2A1, SULT2B1 (PDB accession number: 3CKL, 3BFX, 4IFB, 1Q22), *Arabidopsis thaliana* SOT16 and SOT18 (PDB accession number: 8K9Y, 5MEX), *Streptomyces toyocaensis* Stal (PDB accession number: 2OV8) and *Mycobacterium tuberculosis* glycolipidST. Full alignment was conducted using MAFFT E-INS-i algorithm

	*Hw*SULT| *H. werneckii*	*Fg*SULT|*F. graminearum*	*As*SULT|*A. sydowii*	ST1B1|Human|3CKL	ST1C2|Human|3BFX	ST2A1|Human|4IFB	ST2B1|Human|1Q22	SOT16|*A. thaliana* |8K9Y	SOT18| *A. thaliana*|5MEX	StaL|*S. toyocaensis* |2OV8	GlycolipidST| *M. tuberculosis*
*Hw*SULT| *H. werneckii*	100,0	49,1	16,3	20,3	19,8	19,4	19,7	15,1	14,1	14,5	25,9
*Fg*SULT|*F. graminearum*	49,1	100,0	18,2	19,3	21,2	17,6	17,6	13,9	12,7	14,8	27,3
*As*SULT|*A. sydowii*	16,3	18,2	100,0	27,0	27,9	27,8	20,9	20,5	21,4	12,2	15,9
ST1B1|Human|3CKL	20,3	19,3	27,0	100,0	52,9	36,5	34,3	22,7	21,7	15,7	17,6
ST1C2|Human|3BFX	19,8	21,2	27,9	52,9	100,0	35,3	31,9	23,6	23,4	16,9	16,5
ST2A1|Human|4IFB	19,4	17,6	27,8	36,5	35,3	100,0	44,2	21,3	18,9	16,6	17,1
ST2B1|Human|1Q22	19,7	17,6	20,9	34,3	31,9	44,2	100,0	16,8	16,7	15,7	16,1
SOT16|*A. thaliana* |8K9Y	15,1	13,9	20,5	22,7	23,6	21,3	16,8	100,0	73,1	11,5	13,0
SOT18| *A. thaliana*|5MEX	14,1	12,7	21,4	21,7	23,4	18,9	16,7	73,1	100,0	12,5	12,4
StaL|*S. toyocaensis* |2OV8	14,5	14,8	12,2	15,7	16,9	16,6	15,7	11,5	12,5	100,0	13,4
GlycolipidST| *M. tuberculosis*	25,9	27,3	15,9	17,6	16,5	17,1	16,1	13,0	12,4	13,4	100,0

Figure S2: Percent identity matrix of identified PAPS binding residues between *Hortaea werneckii* UBOCC-A-208029 putative SULT, *Aspergillus sydowii* UBOCC-A- 108050 putative SULT, Human SULT1B1, SULT1C2, SULT2A1, SULT2B1 (PDB accession number: 3CKL, 3BFX, 4IFB, 1Q22), *Arabidopsis thaliana* SOT16 and SOT18 (PDB accession number: 8K9Y, 5MEX), *Streptomyces toyocaensis* Stal (PDB accession number: 2OV8) and *Mycobacterium tuberculosis* glycolipidST. Full alignment was conducted using MAFFT E-INS-i algorithm

	*Hw*SULT| *H. werneckii*	*As*SULT|*A. sydowii*	ST1B1|Human|3CKL	ST1C2|Human|3BFX	ST2A1|Human|4IFB	ST2B1|Human|1Q22	SOT16|*A. thaliana* |8K9Y	SOT18| *A. thaliana*|5MEX	StaL|*S. toyocaensis* |2OV8	GlycolipidST| *M. tuberculosis*
*Hw*SULT|*H. werneckii*	100,0	66,7	72,2	66,7	61,1	66,7	66,7	66,7	50,0	55,6
*As*SULT| *A. sydowii*	66,7	100,0	83,3	77,8	66,7	77,8	72,2	72,2	55,6	72,2
ST1B1|Human|3CKL	72,2	83,3	100,0	94,4	72,2	83,3	72,2	72,2	55,6	72,2
ST1C2|Human|3BFX	66,7	77,8	94,4	100,0	66,7	77,8	72,2	72,2	61,1	66,7
ST2A1|Human|4IFB	61,1	66,7	72,2	66,7	100,0	88,9	72,2	72,2	50,0	55,6
ST2B1|Human|1Q22	66,7	77,8	83,3	77,8	88,9	100,0	77,8	77,8	55,6	66,7
SOT16|*A. thaliana* |8K9Y	66,7	72,2	72,2	72,2	72,2	77,8	100,0	100,0	55,6	55,6
SOT18| *A. thaliana*|5MEX	66,7	72,2	72,2	72,2	72,2	77,8	100,0	100,0	55,6	55,6
StaL|*S. toyocaensis* |2OV8	50,0	55,6	55,6	61,1	50,0	55,6	55,6	55,6	100,0	61,1
GlycolipidST| *M. tuberculosis*	55,6	72,2	72,2	66,7	55,6	66,7	55,6	55,6	61,1	100,0

Table S3: PAPS binding residues. Bold residues were extracted from the three-dimensional structures (PDB). Non-bold residues were inferred from the multiple sequence alignment.

protein																		
*Hw*SULT	49K	50S	51G	52T	53T	54W	100K	102H	121R	129S	205F	239C	241F	265M	267N	268K	269G	270D
*As*SULT	83K	84S	85G	86S	87T	88W	134K	136H	159R	167S	223Y	257V	259F	277F	279R	280K	281G	282K
SULT1B1 (3CKL)	**48K**	**49S**	**50G**	**51T**	**52T**	**53W**	**107K**	**109H**	**131R**	**139S**	**194Y**	**228T**	**230F**	**256F**	**258R**	**259K**	**260G**	**261T**
SULT1C2 (3BFX)	**49K**	50A	**51G**	**52T**	**53T**	**54W**	107K	**109H**	**131R**	**139S**	194Y	**228T**	**230F**	**256F**	**258R**	**259K**	**260G**	**261T**
SULT2A1 (4IFB)	**44K**	**45S**	**46G**	**47T**	**48N**	**49W**	97S	**99H**	**121R**	**129S**	184Y	**218S**	**220F**	245I	**247R**	**248K**	**249G**	**250V**
SULT2B1 (1Q22)	**70K**	**71S**	**72G**	**73T**	**74T**	**75W**	123S	125H	**147R**	**155S**	210Y	**244S**	**246F**	272F	**274R**	**275K**	**276G**	**277V**
SOT16 (8K9Y)	**81K**	**82T**	**83G**	**84T**	**85T**	**86W**	141S	143H	**165R**	**173S**	231Y	**270C**	**272F**	299Y	**301R**	**302K**	**303G**	**304K**
SOT18 (5MEX)	**93K**	**94T**	**95G**	**96T**	**97T**	**98W**	153S	**155H**	**177R**	**185S**	243Y	**282C**	**284F**	311Y	**313R**	**314K**	**315G**	**316K**
StaL (2OV8)	12K	13A	14G	15G	16H	17W	65A	67H	90R	98S	163Y	196C	198L	228F	230G	231K	232G	233G
GlycolipidST	40K	41S	42G	43L	44T	45W	95K	97H	116R	124S	217Y	251A	253L	278F	280R	281R	282G	283G

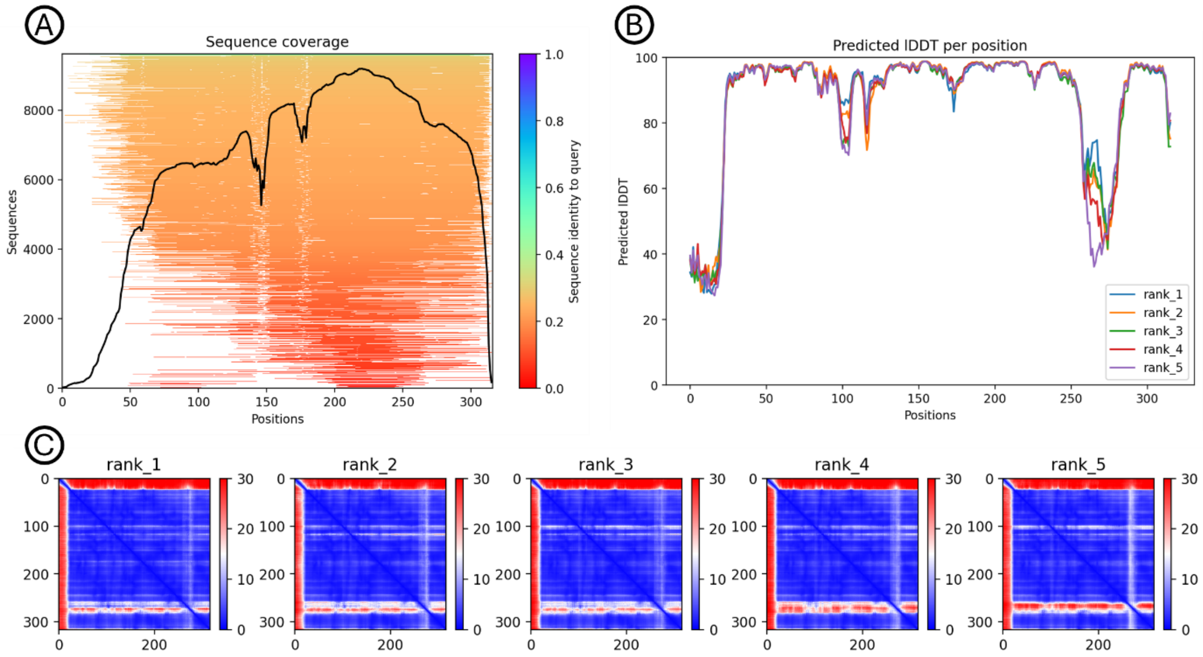

Figure S3: (A) Analysis of sequence coverage of *As*SULT per position. (B) Analysis of the quality confidence of the 5 *As*SULT models pLDDT per position. (C) Plots of the predicted alignment error (PAE) identified per *As*SULT model.

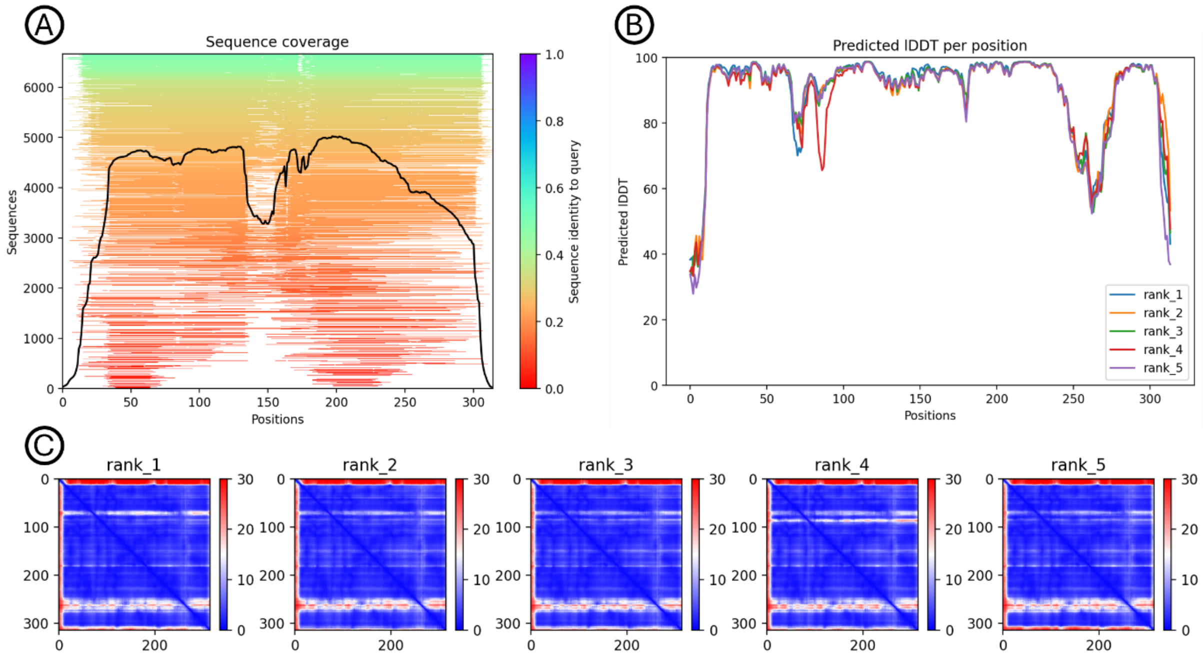

Figure S4: (A) Analysis of sequence coverage of *Hw*SULT per position. (B) Analysis of the quality confidence of the 5 *Hw*SULT models pLDDT per position. (C) Plots of the predicted alignment error (PAE) identified per *Hw*SULT model.

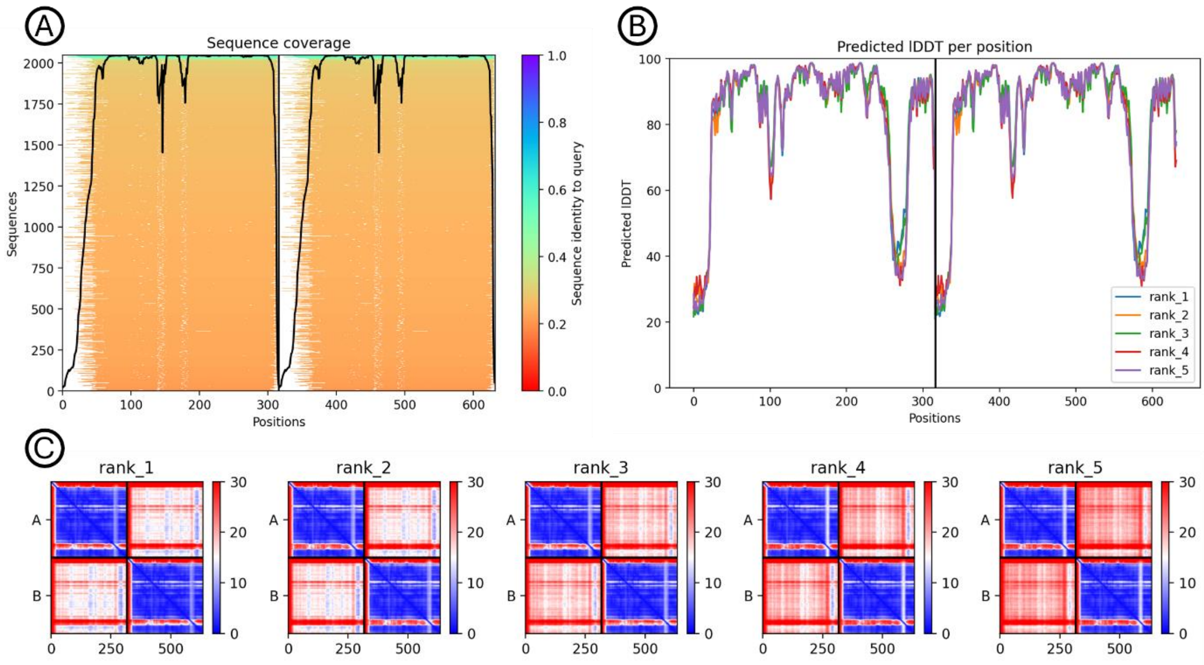

Figure S5: (A) Analysis of sequence coverage of *As*SULT homodimer per position. (B) Analysis of the quality confidence of the 5 *As*SULT homodimer models pLDDT per position. (C) Plots of the predicted alignment error (PAE) identified per *As*SULT homodimer model.

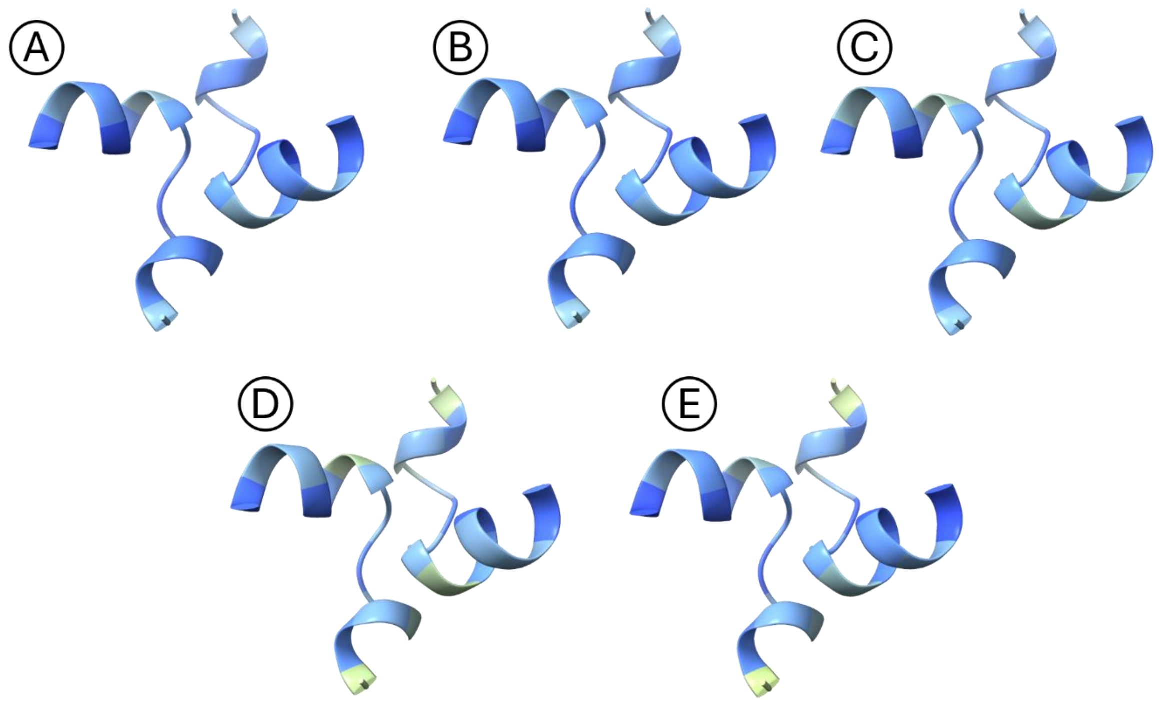

Figure S6: Dimer interface of the *As*SULT homodimer, colored by confidence. A to E correspond to rank 1 to rank 5 model.

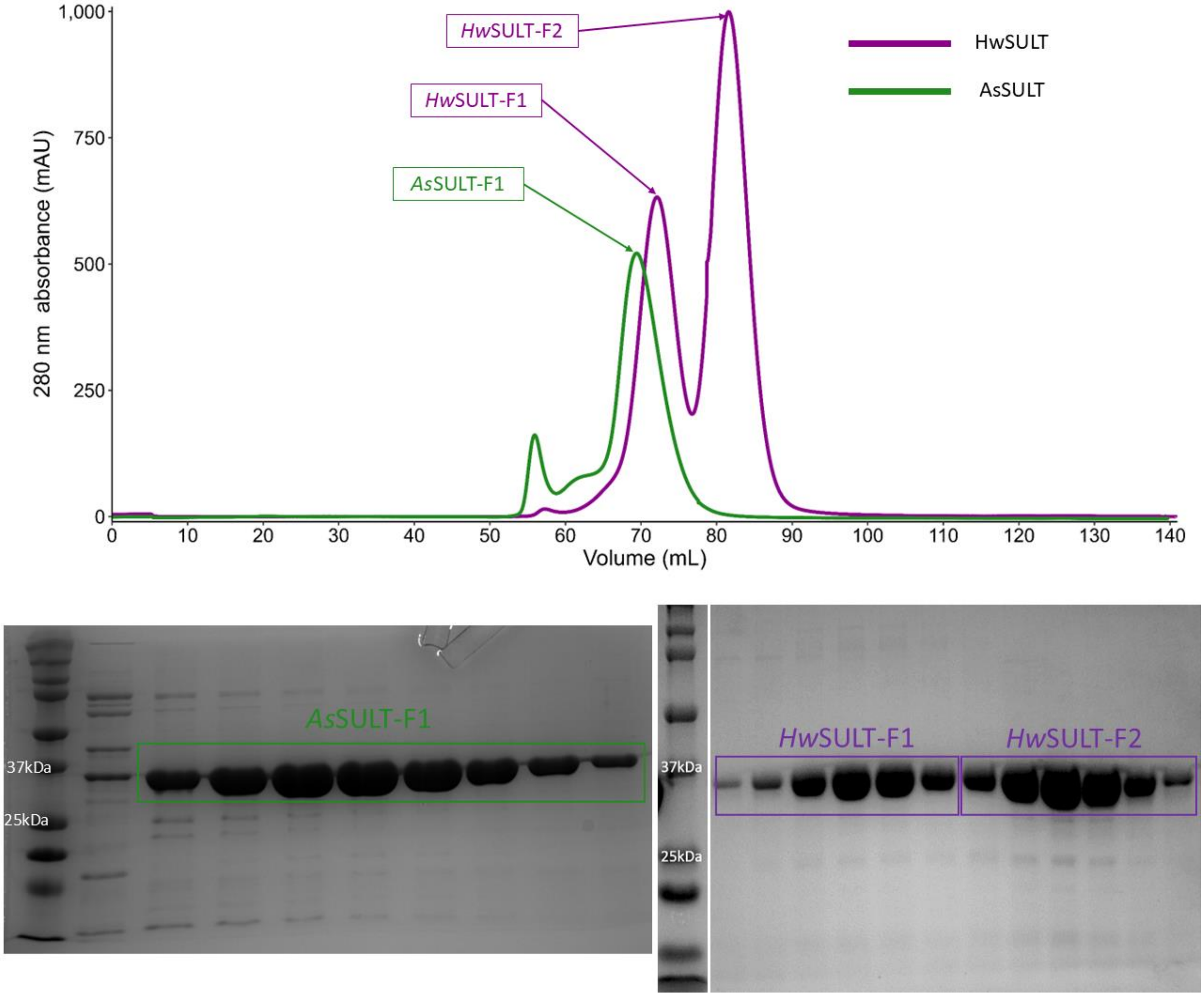

Figure S7: Size Exclusion Chromatogram of *As*SULT and *Hw*SULT with associated 12% SDS-PAGE analysis

Resorcinol sulfate *Hw*SULT

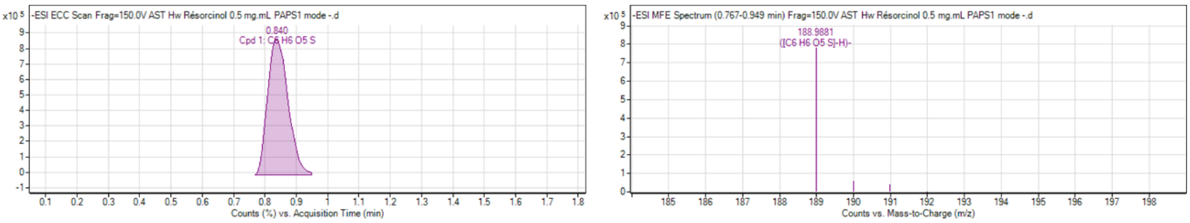

Resorcinol disulfate *Hw*SULT

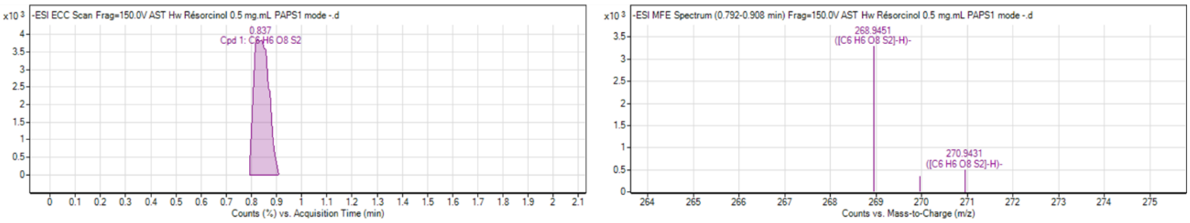

pyrocatechol sulfate *Hw*SULT

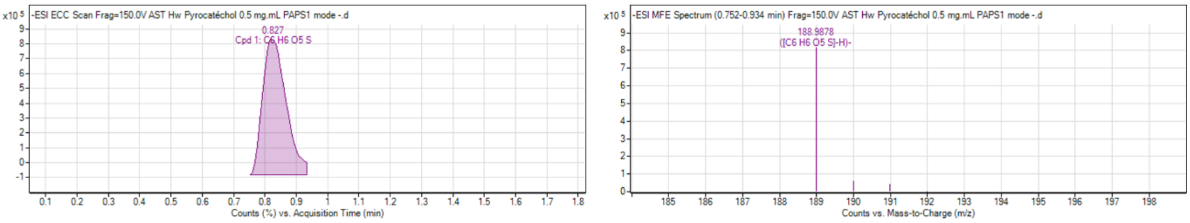

pyrocatechol disulfate *Hw*SULT

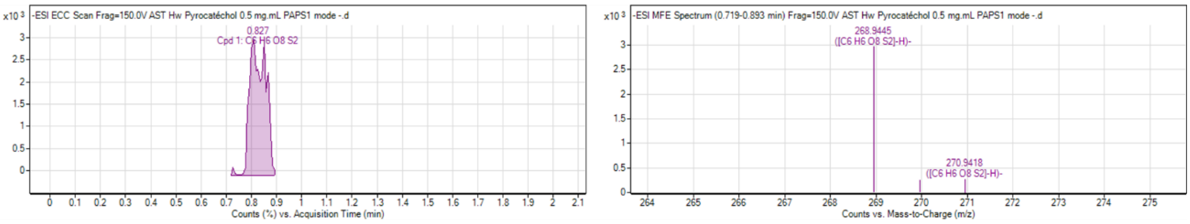

Hydroquinone sulfate *Hw*SULT

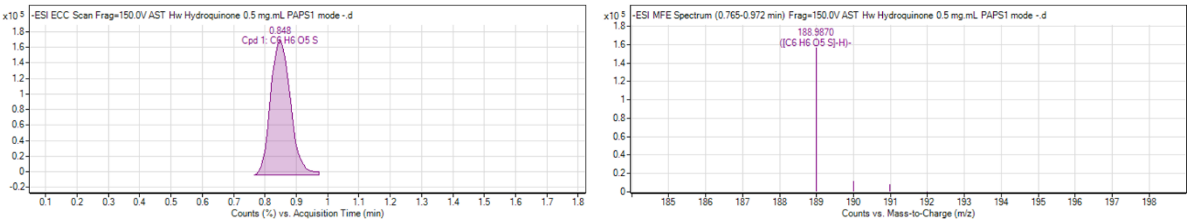

2-aminophenol sulfate *Hw*SULT

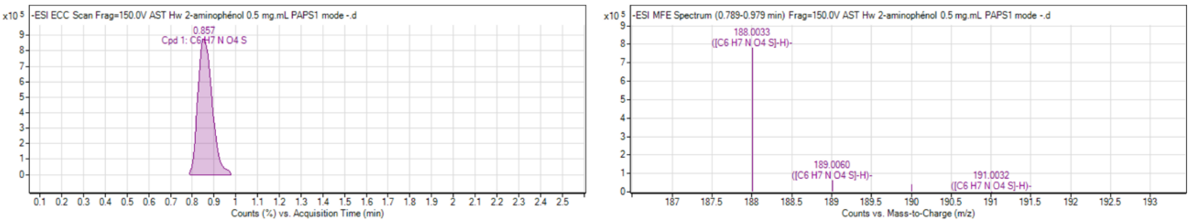

Orcinol sulfate *Hw*SULT

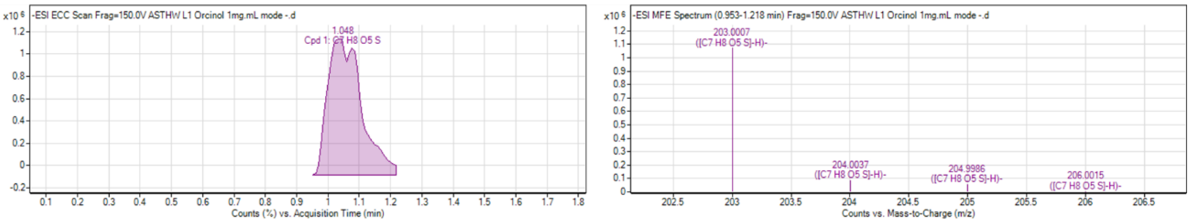

Phloroglucinol sulfate *Hw*SULT

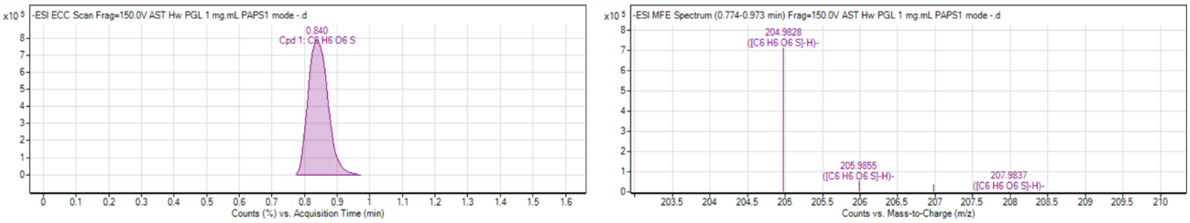

Phloroglucinol disulfate *Hw*SULT

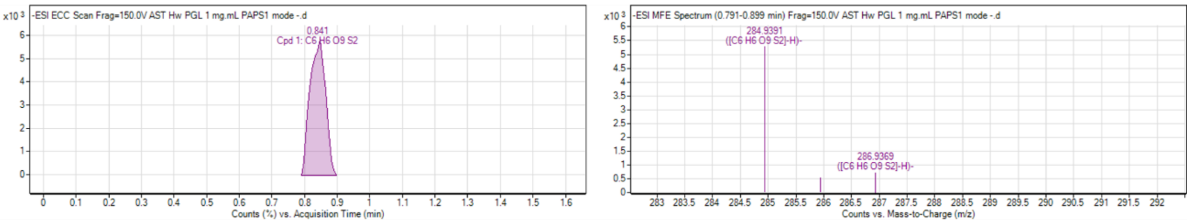

4-methylumbelliferone sulfate *Hw*SULT

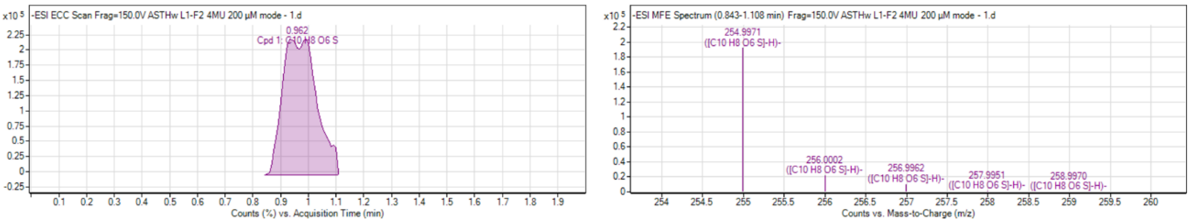

Scopoletin sulfate *Hw*SULT

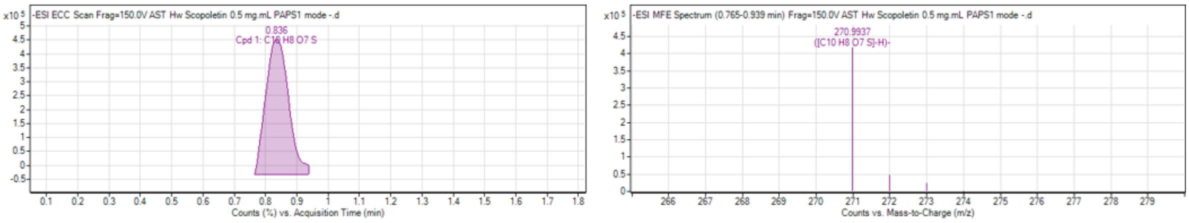

Scopoletin sulfate *As*SULT

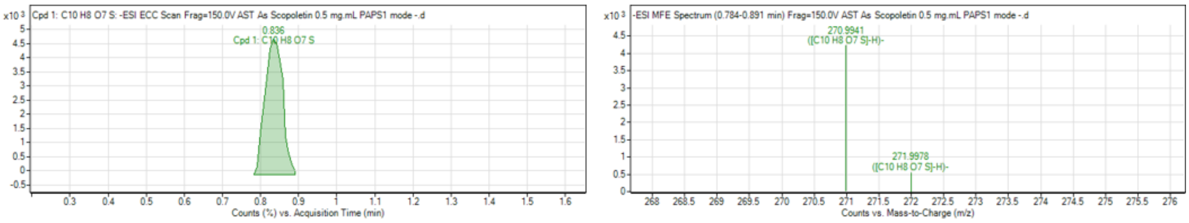

Naringenin sulfate *Hw*SULT

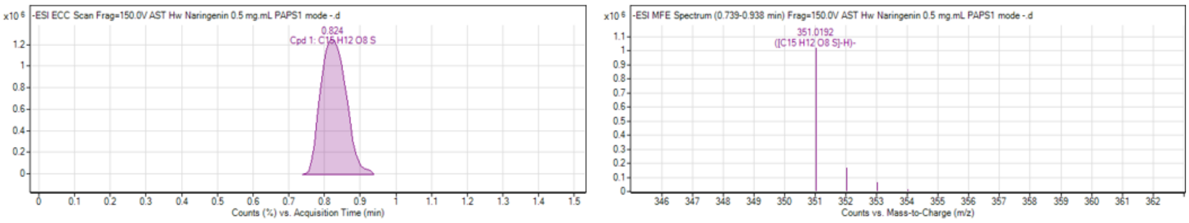

Phloretin sulfate *Hw*SULT

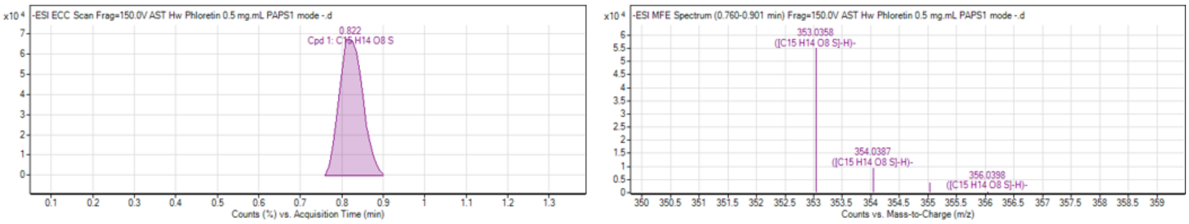

Zearalenone sulfate *Hw*SULT

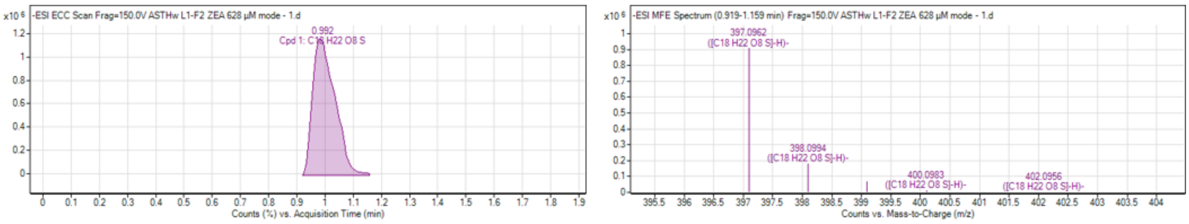

Figure S8: Experimental chromatogram and mass spectra of each molecule sulfated by *Hw*SULT (purple) or *As*SULT (green)

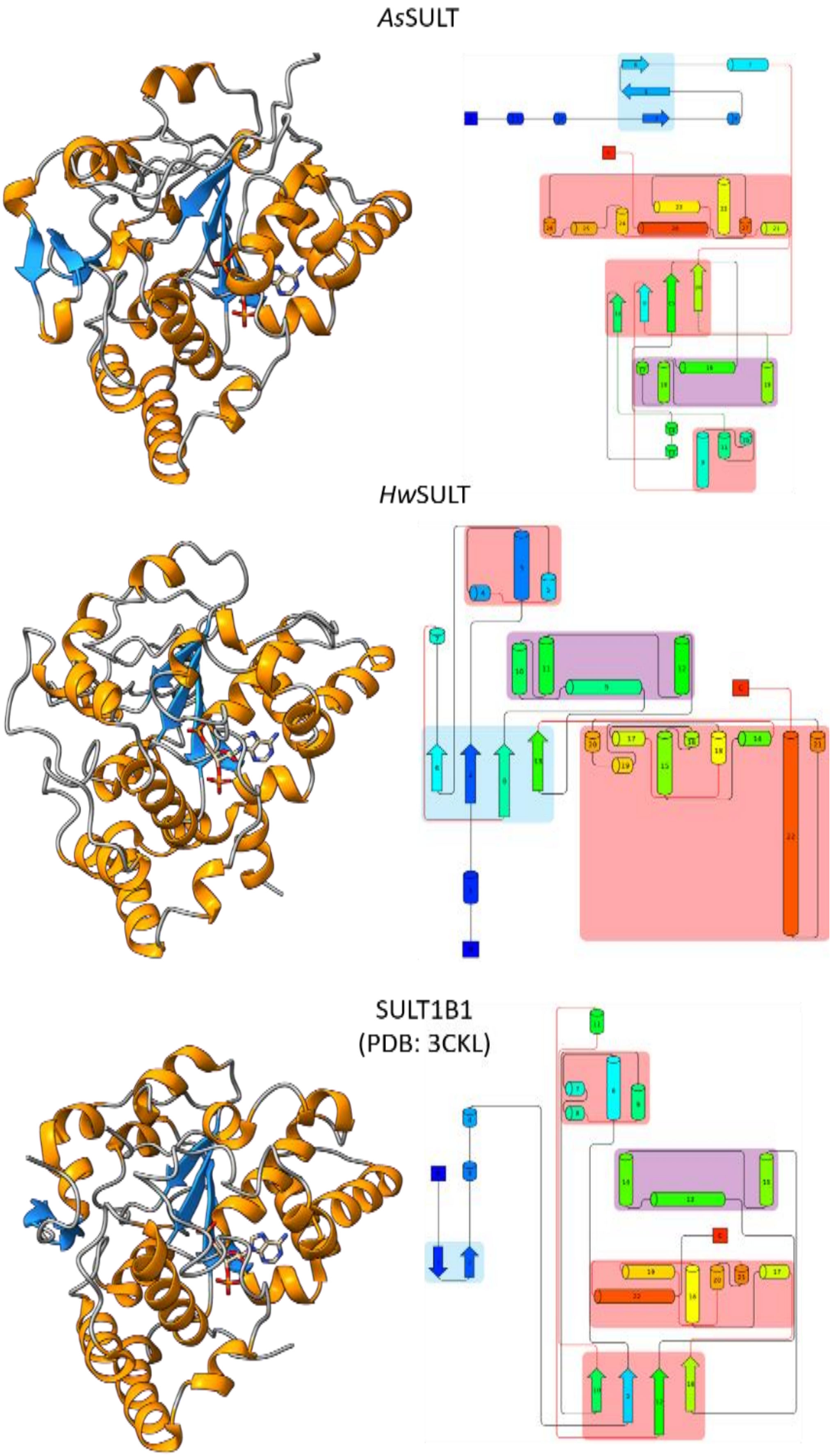

Figure S9: Secondary structure highlights of *Hw*SULT, *As*SULT and SULT1B1 (PDB: 3CKL). Left panel shows three-dimensional structure colored by secondary structures. Α-helix are colored in orange, β-sheet are colored in blue, and loop are colored in grey. Right panel is a topological representation of the secondary structure of each enzyme.

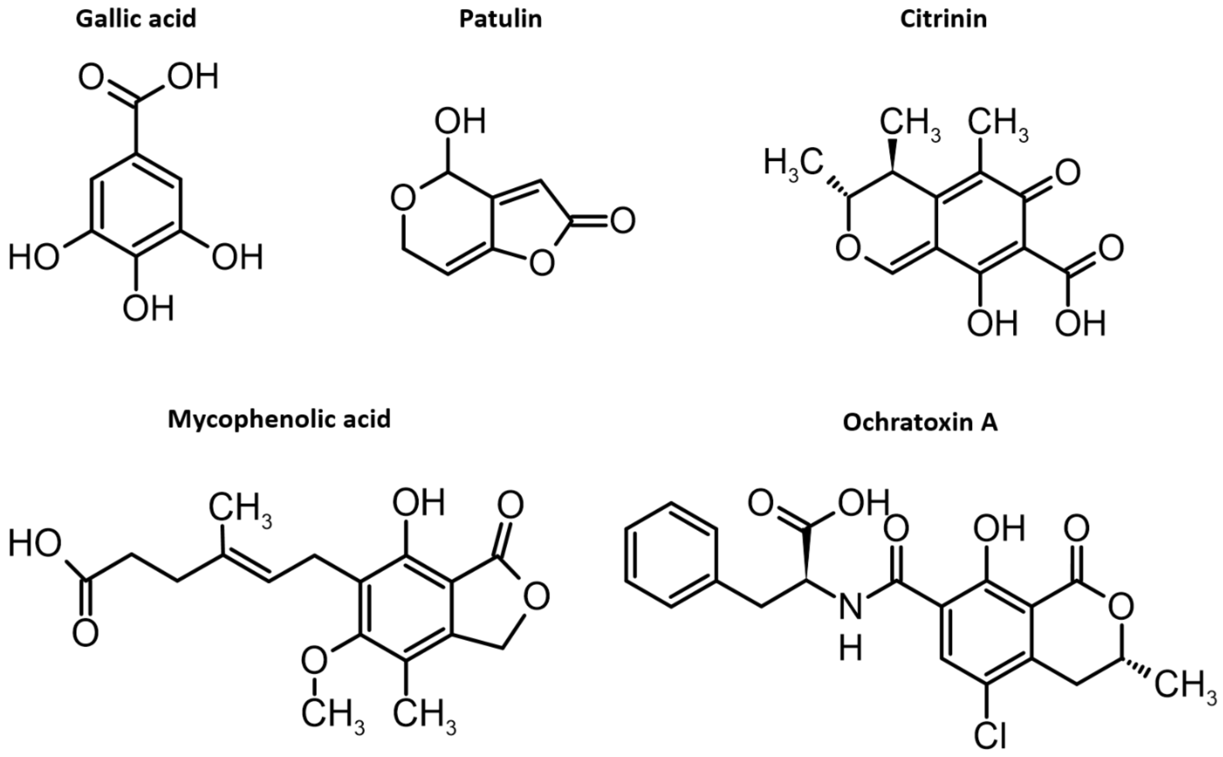

Figure S10: Chemical structures of substrates not sulfated by either *Hw*SULT or *As*SULT

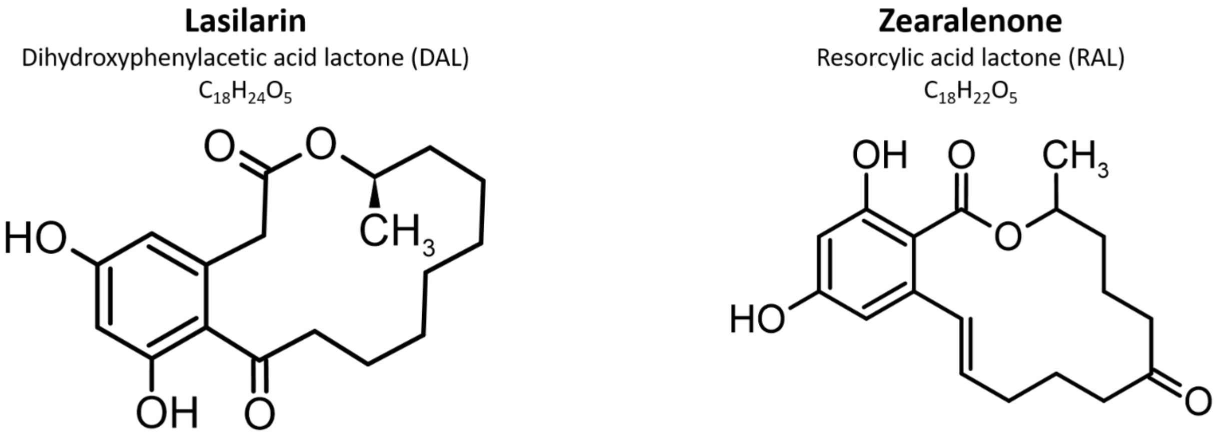

Figure S11: Chemical structures of Lasilarin (Xie et al., 2020) and Zearalenone.

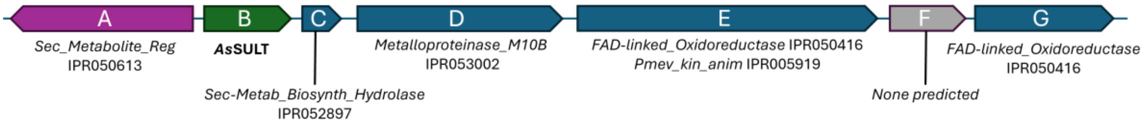

A: IPR050613, *Sec_Metabolite_Reg* **Secondary Metabolite Biosynthesis Regulators**

B: AsSULT, *Sulfotransferase_dom,* IPR000863, PF00685

C: IPR052897, *Sec-Metab_Biosynth_Hydrolase* **Secondary metabolite biosynthesis hydrolase (***AB_hydrolase_1* IPR000073)

D: IPR053002, *Metalloproteinase_M10B* **Zinc-dependent metalloproteinase M10B /**

E: IPR050416 **FAD-linked Oxidoreductases in Biosynthetic Pathways** *FAD-linked_Oxidoreductase*
E: IPR005919 **Higher eukaryotic phosphomevalonate kinase** *Pmev_kin_anim*

F: None predicted

G: IPR050416 **FAD-linked Oxidoreductases in Biosynthetic Pathways** *FAD-linked_Oxidoreductase*

Figure S12: Organization of genes surrounding *As*SULT. Each arrow represents a gene with its corresponding annotation through the interpro analysis.

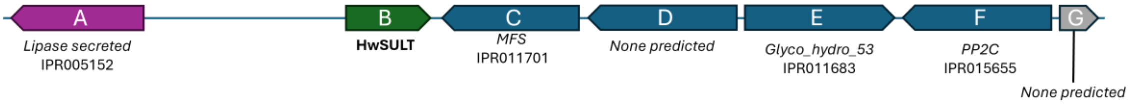

A: IPR005152 **Lipase, secreted**

B: HwSULT, *Sulfotransferase_dom,* IPR000863, PF00685

C: IPR011701 *MFS* **Major facilitator superfamily**

D: None predicted

E: IPR011683 *Glyco_hydro_53* **Glycosyl hydrolase family 53**

F: IPR015655 *PP2C* **Protein phosphatase 2C**

G: None predicted

Figure S13: Organization of genes surrounding *Hw*SULT. Each arrow represents a gene with its corresponding annotation through the interpro analysis.
